# Exploiting dysregulated iron homeostasis to eradicate persistent high-grade serous ovarian cancer

**DOI:** 10.1038/s41420-025-02716-1

**Published:** 2025-09-25

**Authors:** Carmelo Cerra, Madeleine R. C. Tancock, Niko Thio, Ada Koo, AnnRann Wong, Karla J. Cowley, Swati Varshney, Madelynne O. Willis, Kaylene J. Simpson, Australian Ovarian Cancer Study Group, David D. L. Bowtell, Elizabeth L. Christie, David D. L. Bowtell, Elaine Sanij, Elizabeth L. Christie, Richard B. Pearson, Jian Kang, Keefe T. Chan

**Affiliations:** 1 Peter MacCallum Cancer Centre, Melbourne, VIC Australia; 2 Department of Biochemistry and Molecular Biology, Biomedicine Discovery Institute, Monash University, Clayton, VIC Australia; 3 Sir Peter MacCallum Department of Oncology, The University of Melbourne, Melbourne, VIC Australia; 4 Victorian Centre for Functional Genomics, Peter MacCallum Cancer Centre, Melbourne, VIC Australia; 5 Bio21 Mass Spectrometry and Proteomics Facility, The University of Melbourne, Melbourne, VIC Australia; 6 Department of Biochemistry and Pharmacology, The University of Melbourne, Melbourne, VIC Australia; 7 St. Vincent’s Institute of Medical Research, Melbourne, VIC Australia; 8 Department of Medicine, St. Vincent’s Hospital, The University of Melbourne, Melbourne, VIC Australia

**Keywords:** Ovarian cancer, Senescence

## Abstract

Treatments for high-grade serous ovarian cancer (HGSOC) are initially effective but most invariably fail. Although they can successfully suppress the bulk of the tumour cell population, residual cancer cells can enter alternative therapy-resistant cell fates highlighted by proliferative arrest. Understanding the nature of these fates and how cells may resume uncontrolled proliferation will lead to the development of new treatments for HGSOC. In this study, we examine the response of HGSOC cells to standard of care cisplatin chemotherapy and to the RNA Polymerase I transcription inhibitor CX-5461/Pidnarulex, two drugs that elicit a potent DNA damage response and growth arrest. Here, we identify that HGSOC cells exposed to these therapies show multiple hallmarks of therapy-induced senescence (TIS) and derive a core TIS gene expression signature irrespective of genetic background or senescence trigger. Given that TIS is a potentially escapable state, we have performed a focussed drug screen to identify drugs that eradicate senescent HGSOC cells. We identify that therapy-induced senescent HGSOC cells, including those with decreased sensitivity to senolytic drugs that inhibit the pro-survival protein BCL-XL, can be eliminated using drugs that induce ferroptosis, an iron-dependent form of cell death. Mechanistically, we demonstrate that senescent HGSOC cells have altered expression of regulators of iron metabolism leading to intracellular iron overload that underpins this targetable vulnerability. Together, we highlight elevated levels of iron as a TIS biomarker in HGSOC and the potential of inducing ferroptosis to eradicate residual HGSOC cells following initial therapy.

## Introduction

High-grade serous ovarian cancer (HGSOC) is the most common and deadliest gynaecological malignancy. HGSOC is characterised by near universal *TP53* mutations, genomic instability and defective DNA repair mechanisms [1]. Given that HGSOC is often diagnosed in advanced stages, following cytoreductive surgery treatment options are limited and primarily consist of therapies invoking a catastrophic DNA damage and subsequent DNA damage response (DDR). Albeit initially effective, such therapies eventually fail as evidenced by a 60% disease recurrence rate within five years, highlighting the critical unmet need to devise alternative treatment strategies.

While intrinsic and acquired mechanisms of therapy resistance can in part explain treatment failure [2–4], a poorly understood outcome is that following treatment residual surviving cancer cells can enter alternative cell fates typified by a state of cell cycle arrest including drug tolerant persistence [5], diapause-like adaptation [6] and therapy-induced senescence (TIS) [7, 8]. Increasing evidence suggests that these arrest states are transient and escapable, thereby providing a reservoir for cancer recurrence [9, 10]. Recurrent cancer cells have been demonstrated to display pro-tumourigenic features of stemness [11], epithelial-mesenchymal transition (EMT) [12], survival signalling [13], and immune evasion [14].

Drug-tolerant persister cells have recently been observed in HGSOC following chemotherapy with upregulation of the stress-responsive transcription factor *ATF3* and the expression of EMT genes *VIM*, *SNAI1* and *EPCAM* [15]. Depletion of ATF3 could enhance the sensitivity of cancer cells to death by chemotherapy. Residual HGSOC tumours have also shown gene expression signatures consistent with embryonic diapause and displayed overexpression of *CEACAM6*, *CRYAB* and *SOX2*, which may serve as predictive biomarkers of treatment resistance and are prognostic of poor survival [16]. Treatment of HGSOC with Poly-ADP ribose polymerase (PARP) inhibitors was shown to induce a reversible senescence-like phenotype dependent on the cyclin-dependent kinase inhibitor p21 and checkpoint kinase (CHK2) [17]. Furthermore, synthetic lethality was observed with senolytic drugs including ABT-263, which mimics BH3-only proteins to bind and sequester the pro-survival proteins BCL-XL, BCL2 and BCL-W. However, key challenges for progression of ABT-263 through the clinic is on-target platelet toxicity resulting in thrombocytopenia and undefined resistance pathways [18]. Whilst modifications to ABT-263 can mitigate platelet toxicity and overcome resistance, additional treatment strategies through eliminating residual senescent cancer cells via different mechanisms could prove to be beneficial. Therefore, understanding the nature of the cell cycle arrest states following therapy in HGSOC could inform the development of strategies to either prevent cancer cell re-emergence or redirect cancer cells toward death.

Here we examine the fate of HGSOC cells exposed to two drugs that induce a DNA damage response with the aim to define populations of persister cells for therapeutic targeting. Cisplatin is a platinum-based chemotherapy that causes DNA crosslinks and has been used in the treatment of HGSOC [19]. CX-5461/Pidnarulex is an inhibitor of RNA polymerase I transcription and topoisomerase II activity [20] that can induce a p53-independent DNA damage response and is in early phase clinical trials for the treatment of advanced haematological and solid malignancies [21–25]. We identify therapy-induced senescent cells as potential candidates and explore possibilities to eradicate these residual cells following exposure to these therapies.

## Results

### HGSOC cells display a senescent-like phenotype when exposed to DNA-damaging therapy

**Fig. 1:**
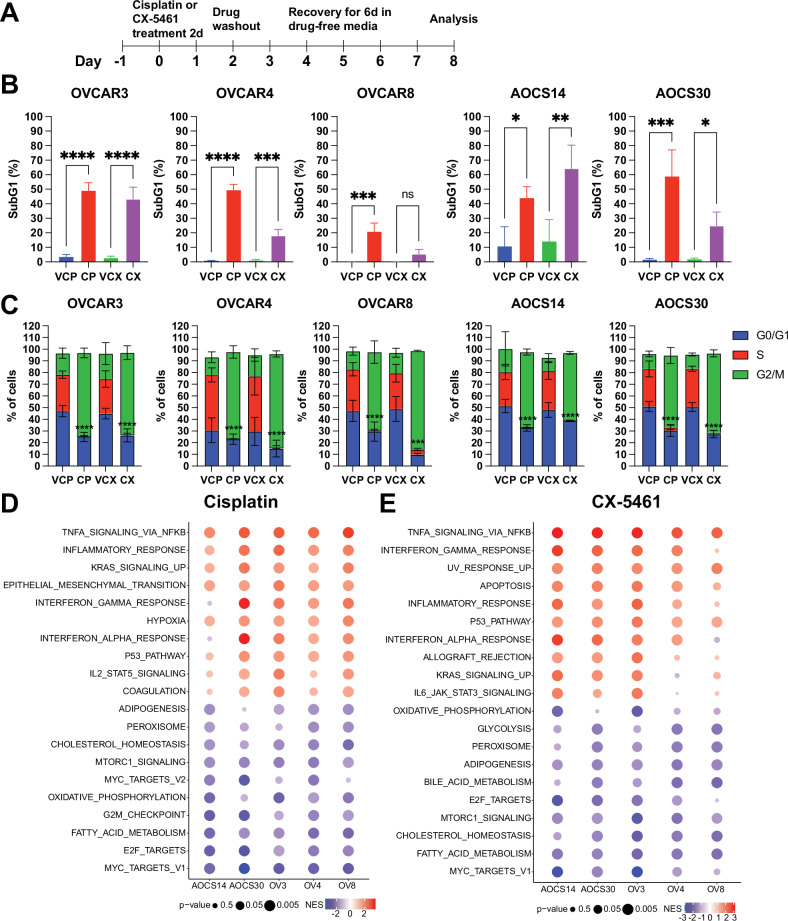
HGSOC cells display a senescent-like phenotype when exposed to DNA damaging therapy. **A** Schematic representation of experimental workflow. **B** Cells were exposed to cisplatin (OVCAR3, 1 µM; OVCAR4, 5 µM; OVCAR8, 10 µM; AOCS14, 1 µM; AOCS30, 3 µM) or CX-5461 (OVCAR3, 1 µM; OVCAR4, 3 µM; OVCAR8, 3 µM; AOCS14, 1 µM; AOCS30, 1 µM) as described in (**A**) and subjected to cell cycle analysis. The percentage of sub-G1 cells is shown as mean ± SEM from n = 3 independent experiments. Statistical analysis was performed using one-way ANOVA with a Šídák’s multiple comparisons test. *p < 0.05; **p < 0.01; ***p < 0.001; ****p < 0.0001; ns not significant. **C** The percentage of cells in G0/G1, S and G2/M phases is shown as mean ± SEM from *n* = 3 independent experiments. Statistical analysis was performed using two**-**way ANOVA with a Tukey’s multiple comparison test and the significance is shown for the S phase population of cells exposed to drug versus their vehicle control (CP vs. VP, CX vs. VCX). ***p < 0.001, ****p < 0.0001. **D**, **E** Bubble plot showing gene set enrichment analysis of Hallmark gene sets (top 10 upregulated and downregulated ranked by mean normalised enrichment score (NES) for all cell lines, with an adjusted p-value < 0.05 for a minimum of two cell lines) for differentially expressed genes between cells treated with (**D**) cisplatin or (**E**) CX-5461 and their respective vehicle controls. NES is shown in the coloured scale. Size of the bubbles is inversely proportional to *p*-value for statistical significance.

We selected three human HGSOC cell lines (OVCAR3, OVCAR4, OVCAR8) and two patient-derived chemo-naïve cell lines from the Australian Ovarian Cancer Study (AOCS14, AOCS30) encompassing a range of different genetic backgrounds in addition to their known mutations in *TP53* [3, 26]. Of note, OVCAR3 displays amplification in *CCNE1*, encoding the cell cycle protein cyclin E1, and is altered in 20% of HGSOC [27]. OVCAR8 has a *KRAS* G12C mutation and hemi-methylated *BRCA1* [28]; AOCS14 harbours a germline *BRCA1* mutation and *RB1* loss [29]. Alterations in *BRCA1* or *BRCA2* in HGSOC include germline and somatic mutations (20%) and hypermethylation of BRCA1 (10%), resulting in homologous recombination dysfunction [27]. To establish a population of residual cells in an arrested state, we exposed cells to cisplatin or CX-5461 or their respective vehicle controls for 48 hours followed by six days of recovery in drug-free medium (Fig. 1A). For each cell line, we optimised drug concentrations that would result in near complete cell cycle arrest. Flow cytometry analysis demonstrated increased cell death in drug-exposed cells as measured by an increase in the sub-G1 population (Fig. 1B). Analysis of the viable 2C-4C population showed a robust G2/M phase arrest, consistent with a p53-independent cell cycle checkpoint (Fig. 1C).

To gain further insight into the cell cycle arrest phenotype, we profiled the transcriptomes of cisplatin- or CX-5461-treated OVCAR3, OVCAR4, OVCAR8, AOCS14, AOCS30 cells and vehicle control cells using 3’ RNA sequencing (RNA-seq). Multi-dimensional scaling analysis demonstrated clustering of the data by each cell line, indicating that even within the same cancer type, patient differences are a major factor dictating TIS gene expression changes (Fig. S1A). In addition, correlation analysis showed pronounced correlation between differentially upregulated or downregulated genes regardless of the treatment tested (Fig. S1B–F), highlighting the possibility to identify gene expression signatures independent of genetic background.

To determine the pathways associated with changes in gene expression in drug- versus vehicle-exposed cells, we performed gene set enrichment analysis focussing on the Hallmark gene sets, which encompass those with well-defined biological processes (Fig. 1D, E). Upregulated genes in cisplatin- and CX-5461-exposed cells were associated with tumour necrosis factor (TNF)-alpha signalling via nuclear factor kappa light chain enhancer of activated B cells (NF-κB), interferon response gamma, interferon response alpha, and inflammatory response. Downregulated genes showed enrichment for early region 2 binding factor (E2F) targets indicative of cell cycle arrest. Cisplatin-exposed cells also showed downregulation of G2/M checkpoint genes, suggesting a greater degree of cell cycle gene repression compared to CX-5461-exposed cells. Cisplatin- and CX-5461-exposed cells displayed downregulation of MYC Targets, a feature observed in cells undergoing a diapause-like adaptation following chemotherapy [6]. In addition, both cisplatin- and CX-5461-treated cells showed downregulation of genes associated with fatty acid metabolism and mTORC1 signalling. We verified activation of the NF-κB signalling pathway by Western blotting (Fig. S1G). Cisplatin and CX-5461 treatment increased the phosphorylation level of the NF-κB subunit p65, along with a reduction in total IκBα protein level. This finding is in line with the well-known role of NF-κB signalling in premature senescence induced by chemotherapy through regulation of the senescence-associated secretory phenotype (SASP) [30].

**Fig. 2:**
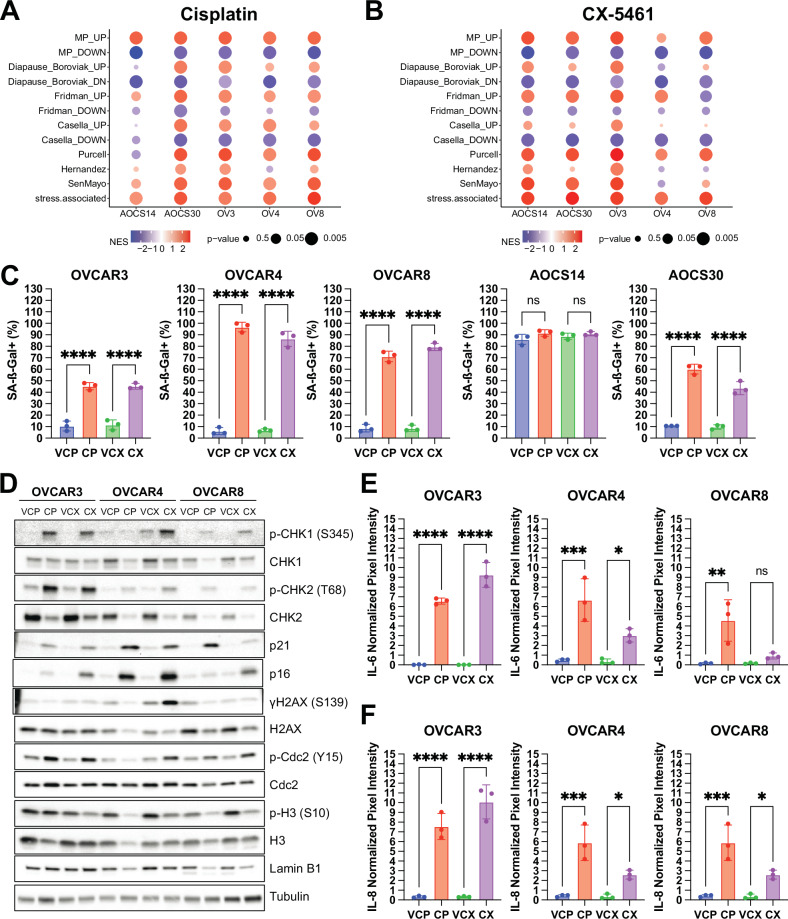
HGSOC cells display a senescent-like phenotype when exposed to DNA damaging therapy. **A**, **B** Bubble plot showing of gene set enrichment analysis of mTOR persister (MP), diapause, senescence (Fridman, Casella, Purcell, Hernandez), and stress-associated gene signatures for differentially expressed genes between OVCAR3 (OV3), OVCAR4 (OV4), OVCAR8 (OV8), AOCS14 and AOCS30 cells exposed to **A** cisplatin or **B** CX-5461 and their respective vehicle controls. Normalised enrichment score is shown in the coloured scale. Size of the bubbles is inversely proportional to *p*-value for statistical significance. **C** The percentage of cells with SA-ß-gal activity as assessed by DDAOG staining is shown as mean ± SEM from *n* = 3 independent experiments. Statistical analysis was performed using one-way ANOVA with a Šídák’s multiple comparisons test. ****p < 0.0001; ns = not significant. **D** Representative Western blots of DNA damage response signaling, cell cycle and senescence markers from OVCAR3, OVCAR4 and OVCAR8 cells exposed to cisplatin or CX-5461 and their respective vehicle controls. Tubulin was probed as a loading control. Quantification of **E** IL-6 **F** IL-8 from conditioned medium isolated from OVCAR3, OVCAR4 and OVCAR8 cells exposed to cisplatin or CX-5461 and their respective vehicle controls is shown as mean ± SEM from *n* = 3 independent experiments. Statistical analysis was performed using one-way ANOVA with a Šídák’s multiple comparisons test. *p < 0.05; **p < 0.01; ***p < 0.001; ****p < 0.0001; ns not significant.

Given that cisplatin and CX-5461 treatment resulted in suppressed MYC target genes and downregulation of mTORC1 signalling, we asked whether cells that display these features could represent those with a form of drug-tolerant persistence [6]. We interrogated a study examining drug-tolerant persistence following mTOR inhibition in pancreatic cancer [31]. The differentially upregulated and downregulated genes following both cisplatin and CX-5461 treatment were strongly enriched for the pancreatic mTOR drug-tolerant persister signatures (MP up) and (MP down), respectively, supporting the observation of increased drug-tolerant persistence upon mTOR inhibition observed in p53-mutant cells [31] (Fig. 2A, B). We also found that cisplatin- and CX-5461-exposed cells displayed transcriptional programs associated with diapause-like adaptation, as evident by four out of five cell lines showing enrichment of genes upregulated in diapause (Diapause_Boroviak_UP) and all showing enrichment of genes downregulated in diapause (Diapause_Boroviak_DN) following treatment (Fig. 2A, B). Our identification that residual cells exposed to cisplatin and CX-5461 have persistence and diapause programs is consistent with the concept that treatment-resistant cells in an arrested state harbour potential vulnerabilities for their elimination [5, 6].

To determine if published senescence gene expression signatures were represented in cisplatin- and CX-5461-exposed HGSOC cells, we performed fast gene set enrichment analysis (FGSEA) of the Fridman, Casella, Purcell, and Hernandez signatures [32–35]. Generally, cells treated with cisplatin displayed a higher degree of enrichment than those treated with CX-5461 for these signatures, showing a greater normalised enrichment score and statistical significance. We also examined the recently described SenMayo geneset, comprising 125 genes encoding for senescence-associated secretory phenotype (SASP) factors [36], transmembrane and intracellular proteins [37]. Both cisplatin- and CX-5461-exposed cells showed robust enrichment for this geneset, which concurs with the improved reliability of this signature as compared with others [37].

A recent longitudinal single-cell RNAseq study of HGSOC in patients following neoadjuvant chemotherapy identified 12 tumour cell clusters including a cluster showing subclonal enrichment of a “stress-associated” gene signature comprising EMT regulators, stemness, pro-survival genes, and NF-κB targets (481 genes in total) [38]. Both cisplatin and CX-5461-exposed cells demonstrated robust enrichment for this stress-associated gene signature (Fig. 2A, B). Taken together, our findings demonstrate that HGSOC cells exposed to DDR therapies harbour transcriptional programs related to drug-tolerant persistence, diapause and a stress-associated state.

A canonical hallmark of cellular senescence is the induction of senescence-associated-beta galactosidase (SA-β-gal) activity [39]. Following cisplatin or CX-5461 treatment, we found a significant increase in SA-β-gal activity as measured by (9*H*-(1,3-Dichloro-9,9-Dimethylacridin-2-One-7-yl) β-D-Galactopyranoside) (DDAOG) positivity (Fig. 2C). Notably, AOCS14 cells had a high basal SA-β-gal activity, which did not markedly increase upon treatment. OVCAR3, OVCAR4 and OVCAR8 cells displayed expression of protein markers of mitotic arrest as demonstrated by increased phosphorylated cdc2 and decreased phosphorylated histone H3 following cisplatin and CX-5461 treatment (Fig. 2D). We also detected markers of persistent DDR as evident by increased levels of phosphorylated checkpoint kinases (CHK)1, CHK2 and histone variant H2AX [40]. Moreover, we observed p53-independent induction of the cyclin-dependent kinase inhibitor p21 and a trend toward a decreased level of the nuclear envelope protein Lamin B1 [41]. Cytokine arrays of conditioned medium showed increased levels of interleukins IL-6 (Fig. 2E) and IL-8 (Fig. 2F), components of the SASP [36]. Collectively, these results demonstrate that HGSOC cells exposed to DNA damage response-inducing therapies exhibit multiple features of therapy-induced senescence (TIS).

### Derivation of a HGSOC TIS gene signature

**Fig. 3:**
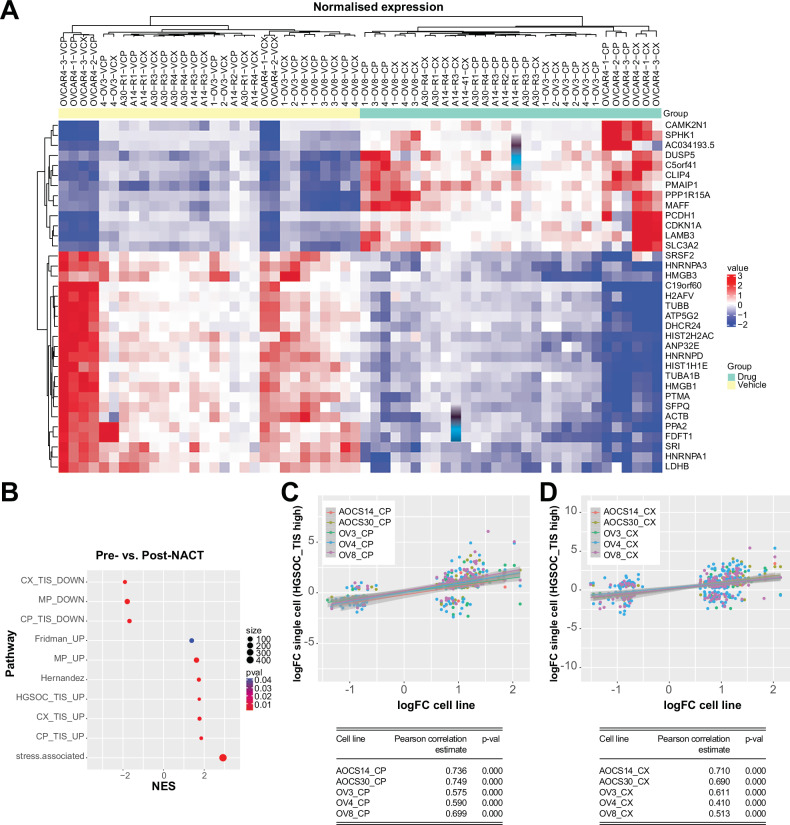
Derivation of a HGSOC TIS gene signature. **A** Heatmap showing core HGSOC TIS signature derived from differentially upregulated and downregulated genes (p-value adjusted < 0.1) between OVCAR3, OVCAR4, OVCAR8, AOCS14 and AOCS30 cells exposed to cisplatin or CX-5461 and their respective vehicle controls. **B** Bubble plot showing gene set enrichment of senescence signatures for differentially expressed genes between pre- and post-NACT single-cell RNAseq samples. Normalised enrichment score is shown with colour scale indicating *p*-value for statistical significance and size indicates number of genes. Correlation plot of differentially expressed genes from OVCAR3, OVCAR4, OVCAR8, AOCS14 and AOCS30 cells exposed to **C** cisplatin or **D** CX-5461 and post-NACT single cell RNAseq samples with a HGSOC_TIS high score > two median absolute deviations compared to all others. Tables indicate Pearson correlation estimate values and their corresponding p-values for statistical significance.

We explored the possibility that we could use our RNA-seq data to derive a TIS gene signature for HGSOC, which would provide an important resource for assessing TIS in tumour cells from patient datasets and for investigating its potential role in therapeutic response or disease persistence. We first derived upregulated and downregulated signatures specific to each drug, which we denoted as CP_TIS and CX_TIS (UP, DOWN). We then intersected the differentially expressed genes across OVCAR3, OVCAR4, OVCAR8, AOCS14 and AOCS30 cells (adjusted *p*-value < 0.01). We identified 13 upregulated genes and 22 downregulated genes common to all cell lines regardless of exposure to cisplatin or CX-5461, which we defined as a HGSOC_TIS gene signature (Fig. 3A).

Notably, within the HGSOC_TIS gene signature, we observed upregulation of *CDKN1A*, encoding for the cyclin-dependent kinase inhibitor p21. This agreed with our observation that p21 protein was induced in a p53-independent manner in response to cisplatin and CX-5461 (Fig. 2D), as has been shown with poly-ADP ribose inhibitors, which can promote TIS [17]. We also found common upregulation of *PMAIP1*, which encodes for NOXA, the endogenous inhibitor of MCL-1, likely reflecting a dependence of therapy-induced senescent cells on BCL-XL for survival and susceptibility to BCL-XL inhibitors. *MAFF*, a member of the small MAF family of bZIP transcription factors, which binds to antioxidant response element-dependent genes, was upregulated. *SLC3A2*, encoding the heavy chain subunit of the system xc- cystine/glutamate antiporter to provide protection against oxidative stress, was also upregulated. E2F target genes encoding the high-mobility group proteins HMGB1 and HMGB3 were downregulated, reflecting altered chromatin structure in TIS [42]. *HNRNPA1*, encoding the heterogeneous nuclear ribonuclear protein A1, was downregulated, suggesting impaired mRNA processing and transport. These data highlight that while cellular functions required for proliferation are impaired, additional salient features such as anti-apoptosis and anti-oxidation contribute to the TIS program in HGSOC. Interestingly, we did not identify commonly upregulated SASP genes, likely due to heterogeneity in the factors expressed for each cell line or treatment. Given the HGSOC_TIS gene signature highlights co-occurrence of oxidative stress and antioxidant response in TIS, we assessed both cytoplasmic and mitochondrial reactive oxygen species (ROS) using 2′,7′-dichlorodihydrofluorescein diacetate (DCFHDA) and MitoSOX, respectively (Fig. S2G, H). As expected, treatment with cisplatin and CX-5461 resulted in significantly elevated ROS levels compared to vehicle controls. We also validated the upregulation of SLC3A2 protein in therapy-induced senescent cells by Western blotting (Fig. S1G). Together, these findings strongly support the notion that oxidative stress is a hallmark of TIS, which is counterbalanced by a coordinated antioxidant response aimed at restoring redox homeostasis.

To determine if we could apply our TIS gene signature to clinical data, we examined single cell RNA-seq data from 11 HGSOC patients having matched pre- and post-treatment samples with neoadjuvant chemotherapy (NACT, GSE165897) (Fig. S2A, B) [38]. Focussing on the tumour cell population, we applied pseudobulk analysis to identify differentially expressed genes pre- and post-NACT treatment for each patient. Gene set enrichment analysis of differentially upregulated genes showed enrichment for Fridman_UP, Hernandez, MP_UP and stress-associated signatures. Moreover, we found significant enrichment for the HGSOC_TIS_UP signature, indicating its utility for delineating senescent cells in a post-NACT setting and potential for exploring senotherapeutic approaches (Fig. 3B). Further supporting this, we determined a positive correlation between post-treatment tumour cells showing a high HGSOC_TIS_UP signature score in this dataset [38] as well as another external single cell RNAseq dataset [43]. In addition, we determined there is a positive correlation between the level of differential gene expression for genes in the HGSOC_TIS signature in post-treatment patient samples and our cell lines (Figs. 3C, D and S2A–F).

### Strategies to eliminate therapy-induced senescent HGSOC cells

Given that residual therapy-induced senescent cells may eventually re-enter the cell cycle to cause disease recurrence, intense efforts have been directed toward eradicating them. Therefore, we sought to understand the potential vulnerabilities of therapy-induced senescent HGSOC cells. The HGSOC_TIS gene expression signature indicated upregulation of *PMAIP1* encoding for NOXA, which inhibits the pro-survival protein MCL-1 and has been shown to correlate with increased reliance on BCL-XL for survival [44]. Consistent with this, OVCAR8 cells showed an increased level of BCL-XL in cisplatin- and CX-5461-induced senescent cells (Fig. S3A). We further confirmed that CRISPR/Cas9-mediated knockout of *BCL2L1* (encoding BCL-XL) could reduce viability of senescent OVCAR8 cells (Fig. S3B). We generated dose-response curves of ABT-263 as well as other BCL-XL inhibitors A-1155463 and ABT-737 [45, 46]. To determine the sensitivity of the drugs for each treatment condition, we quantified the area under the curve (AUC) (Table S1). The BCL-XL inhibitors showed selectivity for compromising the viability of cisplatin- and CX-5461-induced senescent cells as compared with proliferating cells (Fig. S3C–E). As exceptions to this, OVCAR4 cells exposed to CX-5461 showed markedly decreased sensitivity to ABT-263 (AUC = 0.716) as compared with proliferating cells (AUC = 0.915) (Fig. S3E), and OVCAR4 and OVCAR8 CX-5461-induced senescent cells showed resistance to ABT-737 (Fig. S3C, E). The benign fallopian tube epithelial cell line FT282 also demonstrated preferential reduction in cell number upon treatment with the BCL-XL inhibitors for cells exposed to cisplatin and CX-5461 tested versus proliferating cells (Fig. S3F). Collectively, these data in ovarian cancer are consistent with that of previous studies demonstrating that the increased activity of anti-apoptotic proteins following treatment with DDR therapies promotes cell survival [45, 47, 48].

**Fig. 4:**
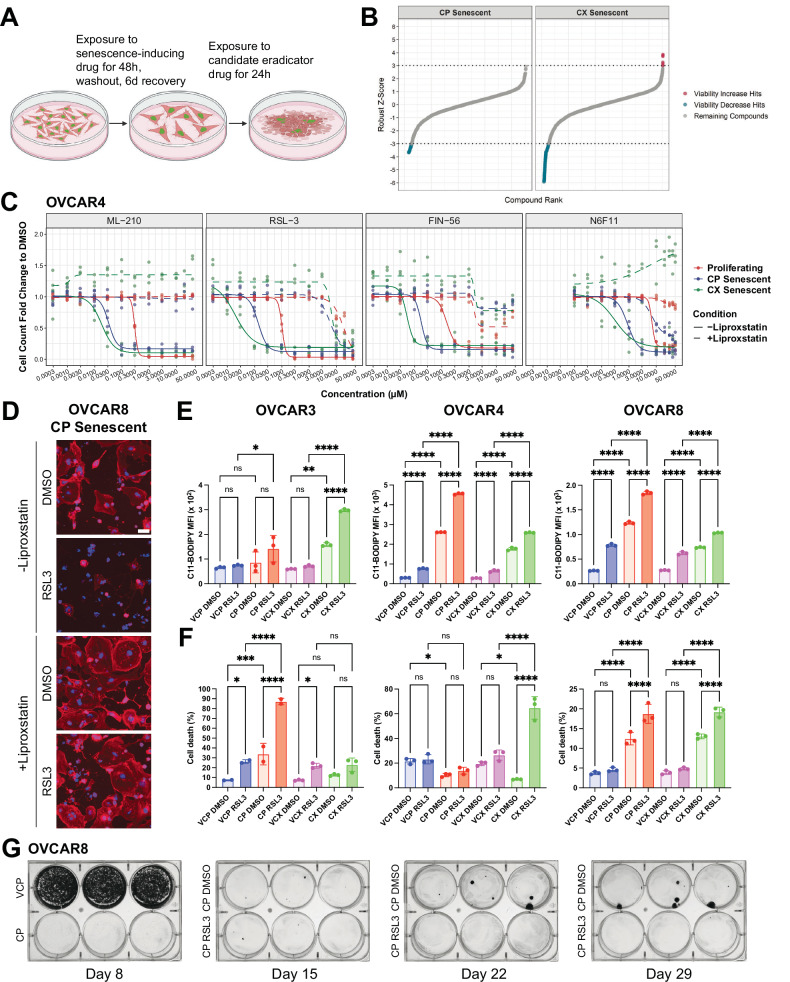
A compound screen for elimination of therapy-induced senescent HGSOC cells. **A** Schematic representation of primary drug screen design was created with Biorender.com. **B** Waterfall plot of Z-scores for 1131 compounds evaluated in cisplatin (CP) and CX-5461 (CX) senescent cells. **C** Dose–response curves for ferroptosis inducers ML-210, RSL3, FIN56 and N6F11. Dashed lines indicate dose–response curves with the inclusion of 1 μM liproxstatin. **D** Representative fluorescent images of OVCAR8 CP TIS cells exposed to RSL3 in the absence or presence of 1 μM liproxstatin. DAPI staining was used to mark nuclei and rhodamine-phalloidin for cell morphology. Cells were only considered if they were positive for DAPI and phalloidin staining. Scale bar = 50 µm. **E** Quantification of median fluorescence intensity (MFI) of lipid peroxidation as measured by C11-BODIPY staining is shown as mean ± SEM from *n* = 3 independent experiments. Statistical analysis was performed using one-way ANOVA with a Šídák’s multiple comparisons test. *p < 0.05; **p < 0.01; ****p < 0.0001; ns not significant. **F** Quantification of the percentage cell death as measured by fixable NIR staining is shown as mean ± SEM from *n* = 3 independent experiments. Statistical analysis was performed using one-way ANOVA with a Šídák’s multiple comparisons test. *p < 0.05; ***p < 0.001; ****p < 0.0001; ns not significant. **G** Representative images of colony formation assay of OVCAR8 cells treated with cisplatin for 48 h, followed by washout. At six days post-washout, cells were treated with control or 100 nM RSL3 for 24 h, then imaged weekly for an additional two weeks.

Given the variability in response to BCL-XL inhibitor-based senolytics depending on cell line or treatment, we sought to identify other cell-intrinsic death pathways we could target to eliminate senescent HGSOC cells. We performed a primary drug screen containing 1131 compounds (a sub-library of apoptosis-related compounds from the Compounds Australia Open Access drug library) (Fig. 4A, B). OVCAR8 cells expressing a histone H2B-green fluorescent protein (H2B-GFP) were initially treated with cisplatin or CX-5461, the senescent cells were then exposed to these compounds at doses of 1 μM or 5 μM for 24 h, and cell number was assessed via 4′,6-diamidino-2-phenylindole (DAPI) staining of nuclei along with a rhodamine-phalloidin, a filamentous actin marker of cell morphology (Tables S2 and S3). On each 384-well plate we used positive control drugs at increasing doses (Fig. S4A, B), which included the BCL-XL inhibitors A-1155463, ABT-263, and ABT-737, which we demonstrated could preferentially kill senescent cells (Fig. S3C–E and Table S1). We also included a non-specific kinase inhibitor staurosporine that can induce apoptosis in proliferating cells in a p53-independent manner [49]. We incorporated the anti-proliferative drug mitomycin, which we expected to have minimal effect in non-dividing cells. As expected, the BCL-XL inhibitors dose-dependently reduced senescent cell numbers (Fig. S4A, B). Staurosporine treatment also resulted in robust levels of cell death, despite previous studies showing that senescent cells typically are resistant to this drug [50]. By stark contrast, mitomycin even at its highest concentration tested (100 μM) failed to eliminate cisplatin- or CX-5461-senescent cells, highlighting their exceptional resistance to killing by anti-proliferative agents.

We identified 59 hit compounds that decreased viability by at least 84% in cisplatin senescent cells and 85 compounds by at least 26% in CX-5461 senescent cells, corresponding to a robust Z-score ≤ −3 (Fig. 4B). We determined an overlap of 52 compounds that decreased viability in both cisplatin and CX-5461 senescent cells (Fig. S4C). In a secondary screen, we generated five-point dose-response curves of these compounds for senescent and, in parallel, proliferating cells (Fig. S4C and Table S4).

Consistent with our expectation that BCL-XL inhibitors would preferentially kill senescent cells over proliferating cells, this occurred upon exposure to the dual BCL-2/BCL-XL inhibitor AZD4320 albeit to a greater extent in cisplatin- versus CX-5461-induced senescent cells. This agrees with the variable responses to BCL-XL inhibitors we observed during our screen optimisation. Interestingly, the WEE1 inhibitor PD0166285 killed proliferating cells with an IC50 = 1.819 µM, whereas cisplatin- and CX-5461-induced senescent cells showed IC50 = 506 nM and 361 nM, respectively. This is in accordance with the reported ability of WEE1 inhibition in forcing mitotic entry from S-phase arrested p53-mutant cells [51].

The second mitochondria-derived activator of caspases (SMAC) mimetic AZD5582 [52], which acts by antagonising inhibitors of apoptosis proteins, could also preferentially eradicate cisplatin- and CX-5461-induced senescent cells (AUC = 0.217, AUC = 0.428, respectively) versus proliferating cells (AUC = 0.736). Collectively, our results show that in addition to senescent cell death upon exposure to BCL-XL inhibitors, we could identify drugs acting via different mechanisms that could target senescent cancer cells.

### Therapy-induced senescent HGSOC cells are sensitive to ferroptosis induction

Strikingly, OVCAR8 cells showed remarkable sensitivity to CIL56, ML-210 and RSL3, which was further enhanced when cells were rendered senescent by cisplatin or CX-5461 (Table S4). These drugs act by inducing ferroptosis, an iron and lipid peroxidation-dependent mechanism of cell death [53].

To validate the effectiveness of ferroptosis inducers in killing senescent cells, we performed a tertiary screen of proliferating and therapy-induced senescent OVCAR3, OVCAR4, and OVCAR8 cells. We exposed cells to ferroptosis-inducing drugs that have different mechanisms of action converging on inhibition of glutathione peroxidase (GPX4), a critical lipid peroxide detoxification enzyme: ML-210, RSL3, FIN56 and N6F11 (Figs. 4C, S5A, B and Table S5). The active form of ML-210, requiring cellular conversion to JKE-1674, and RSL3 are direct inhibitors of GPX4 [54, 55]. FIN56, a newer analogue of CIL56, promotes GPX4 degradation and activates squalene synthase, an enzyme important in cholesterol biosynthesis [56]. N6F11 activates the E3 ubiquitin ligase tripartite motif containing 25 (TRIM25) to mediate GPX4 degradation and has been shown to be selective for cancer cells [57].

In OVCAR4 and OVCAR8 cells, these drugs showed marked selectivity for cisplatin- and CX-5461-induced senescent cells versus proliferating cells (Figs. 4C and S5A). We noted a dramatic cell shrinkage, characteristic of ferroptosis, in senescent HGSOC cells exposed to RSL3 (Fig. 4D). Despite variability due to low cell numbers following treatment, OVCAR3 therapy-induced senescent HGSOC cells also showed sensitivity particularly to ML-210 and RSL3 (Fig. S5B). On the other hand, FT282 fallopian tube epithelial cells exposed to cisplatin and CX-5461 showed resistance to erastin, ML-210 and RSL3, indicating sensitivity to these drugs is preferential to therapy-induced senescent HGSOC cells (Figs. S5C and Table S6). Co-treatment of OVCAR3, OVCAR4 and OVCAR8 cells with the ferroptosis inhibitor liproxstatin could abrogate cell death in response to ML-210, RSL3, FIN56 and N6F11, highlighting that residual senescent HGSOC cells following treatment can be eradicated with drugs that induce ferroptosis (Figs. 4C, D, S5A, B, and Table S5).

To further validate the ability of senescent HGSOC cells to undergo ferroptosis, we measured lipid peroxidation, a hallmark of ferroptotic cell death (Fig. 4E). We exposed proliferating or senescent OVCAR3, OVCAR4 and OVCAR8 cells to 1 µM RSL3 for six hours followed by flow cytometry analysis of C11-BODIPY, a fluorescent lipid peroxidation probe. Cisplatin and CX-5461 therapy-induced senescent cells showed a higher basal level of lipid peroxidation than their vehicle-treated counterparts, indicative of ferroptotic priming. Whilst both proliferating and senescent HGSOC cells showed significant increases in lipid peroxidation upon exposure to RSL3, this was dramatically increased in senescent cells, indicating their sensitisation to ferroptosis. Where we did not observe a significant increase in lipid peroxidation, as for OVCAR3 cells rendered senescent by cisplatin, we instead observed a marked increase in cell death already within this time period (Fig. 4F). To assess dynamic changes in mitochondrial function, we examined the expression of mitochondrial oxidative phosphorylation complex proteins by Western blotting. Overall, no consistent alterations were observed in therapy-induced senescent cells, or those treated with either ABT263 or RSL3 (Fig. S6A). We next evaluated mitochondrial membrane potential (MMP) using the fluorescent probe tetramethylrhodamine ethyl ester (TMRE) (Fig. S6B). MMP was increased in senescent cells, particularly in OVCAR4 and OVCAR8 cells, while no significant change was detected in CX-5461-induced senescent OVCAR3 cells. Mitochondrial hyperpolarisation has previously been reported to occur during cysteine deprivation-induced ferroptosis, likely due to enhanced mitochondrial electron transport chain activity, which promotes ROS production and lipid peroxidation [58]. In line with this, the observed MMP elevation in therapy-induced senescent cells coincided with increased mitochondrial ROS levels (Fig. S2G) and lipid peroxidation (Fig. 4F), suggesting acquisition of ferroptosis-associated metabolic features. Subsequent treatment with ABT-263 reduced MMP, consistent with its known action on mitochondrial outer membrane permeabilization and apoptosis induction [59]. In contrast, RSL3 had no significant effect on MMP, in accordance with its mechanism of inducing lipid peroxidation through GPX4 inhibition independently of MMP disruption. Collectively, these findings support that therapy-induced senescent HGSOC cells exhibit ferroptosis-associated features, including lipid peroxidation and mitochondrial hyperpolarisation, rendering them susceptible to GPX4 inhibition-induced ferroptosis.

To examine the impact of TIS followed by a “hit-and-run” dose of ferroptosis inducer as a new approach to eradicate senescent cells, we performed a clonogenic survival assay of OVCAR8 cells (Fig. 4G). Cells exposed to cisplatin underwent TIS as expected but showed the emergence of colonies two to three weeks post treatment. Remarkably, treatment with 100 nM RSL3 for 24 h following senescence induction prevented colony emergence, highlighting the vulnerability of senescent HGSOC cells to ferroptotic cell death.

### Therapy-induced senescent HGSOC cells display altered iron homeostasis

To understand the mechanisms underpinning therapy-induced senescent HGSOC cell sensitivity to ferroptosis inducers, we first examined the expression of iron metabolism genes [60] in our RNA-seq data. Unexpectedly, despite global changes in gene expression following cisplatin or CX-5461 treatment, we did not find any marked changes in the expression of these genes (Fig. S7). We therefore posited that changes in the proteome, including those that regulate iron metabolism, may provide an alternative explanation for the increased ferroptosis sensitivity of senescent HGSOC cells. Label-free proteomics of OVCAR3, OVCAR4, OVCAR8, AOCS14 and AOCS30 cells and liquid chromatography mass spectrometry LC-MS/MS analysis enabled the detection of approximately 5000 proteins in control and treated cells, and determination of differentially expressed proteins (Fig. S8A and Table S7). Pathway enrichment analysis of differentially upregulated proteins in cisplatin and CX-5461 therapy-induced senescent HGSOC cells vs. control showed significant enrichment of pathways involved in cellular metabolism (TCA cycle, 2-oxocarboxylic acid, carbon) and vesicular transport (SNARE interactions, and phagosome, lysosome) (Fig. S8B). Downregulated proteins in TIS were associated with pathways regulating mRNA translation (ribosome, ribosome biogenesis, RNA polymerase) and DNA repair (DNA replication, mismatch repair, pyrimidine metabolism) (Fig. S8B).

**Fig. 5:**
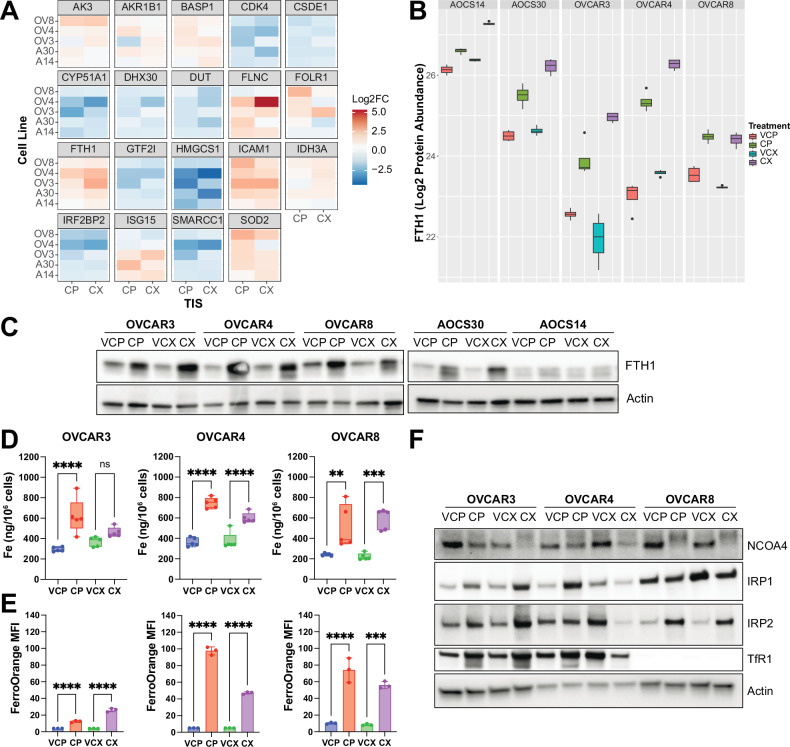
Iron homeostasis is dysregulated in therapy-induced senescent HGSOC cells. **A** Heatmaps of most commonly differentially regulated proteins from proteomics analysis of OVCAR3, OVCAR4, OVCAR8, AOCS14 and AOCS30 cells exposed to cisplatin or CX-5461 and their respective vehicle controls, as determined by number of drug-treated vs. control samples showing fold change > 1.5 or < −1.5. **B** Quantification of FTH1 protein abundance from *n* = 4 independent experiments is shown as mean ± SD. **C** Western blots of FTH1 protein expression. Actin was probed as a loading control. **D** Quantification of total iron content from ICP-MS analysis of OVCAR3, OVCAR4 and OVCAR8 cells exposed to cisplatin or CX-5461 and their respective vehicle controls is shown as mean ± SEM from n = 4 replicates. Statistical analysis was performed using one-way ANOVA with a Šídák’s multiple comparisons test. **p < 0.01; ***p < 0.001; ****p < 0.0001; ns not significant. **E** Quantification of free labile iron as measured by FerroOrange median fluorescence intensity is shown as mean ± SD from *n* = 3 replicates. Statistical analysis was performed using one-way ANOVA with a Šídák’s multiple comparisons test. ***p < 0.001; ****p < 0.0001. **F** Representative Western blots of key regulators of iron homeostasis. Actin was probed as a loading control.

Strikingly, upon examining the most commonly differentially regulated proteins across the cell lines irrespective of the senescence inducer, we identified the ferritin heavy chain (FTH1), a master regulator of iron storage, to be significantly upregulated (fold change (FC) > 1.5) (Fig. 5A, B and Table S7) [61]. In senescent cells, we also observed significant upregulation of superoxide dismutase (SOD2), which plays a critical role in protecting against superoxide radicals in the mitochondria [62] (Fig. 5A and Table S7). Moreover, we noted upregulation of intercellular adhesion molecule (ICAM-1), a common component of the SASP (Fig. 5A and Table S7). Western blot analysis confirmed the upregulation of FTH1 and SOD2 protein in therapy-induced senescent HGSOC cells (Figs. 5C and S8C). In OVCAR3 and OVCAR8 cells, increased FTH1 levels coincided with the induction of glutathione peroxidase GPX4 protein expression, a key ferroptosis inhibitor (Fig. S8D) [55]. Moreover, proteomics analysis of primary, recurrent and end-stage samples from HGSOC patients [2], demonstrated significant correlation between FTH1 and SOD2 protein levels in primary and end-stage samples (Fig. S8E). Immunohistochemistry staining of tissue microarrays also demonstrated a trend toward FTH1 accumulation from primary to end-stage tumours in individual patients (Fig. S8F).

Given the increased FTH1 levels in senescent cells and the role of FTH1 in regulating iron storage, we compared the total cellular iron content in senescent vs. proliferating cells using inductively coupled plasma mass spectrometry. Senescent HSGOC cells had significantly increased levels of total iron content across all three HGSOC cell lines following treatment with either cisplatin or CX-5461 (Fig. 5D). In contrast, changes of other metal ions including magnesium (^24^Mg), calcium (^44^Ca), manganese (^55^Mn), cobalt (^59^Co), nickel (^60^Ni), copper (^63^Cu), zinc (^66^Zn) and cadmium (^111^Cd) were variable despite accumulation of calcium and copper observed in other senescent or cancer models [63, 64], highlighting that elevated intracellular iron levels is a common feature of therapy-induced senescent HGSOC cells (Fig. S9). Intriguingly, the amount of “free” labile iron in senescent cells was also dramatically increased, suggesting that iron was in profound excess (Fig. 5E). To attempt to reconcile these findings, we therefore examined the levels of key regulators of iron homeostasis (Fig. 5F). The transferrin receptor (TfR1) is the major mediator of Fe^3^^+^-transferrin uptake. Furthermore, the iron regulatory proteins (IRPs) are key mediators of iron under conditions of iron deficiency or proficiency. In iron-deficient conditions, IRPs bind to an iron-response element in the 5’ untranslated region of ferritin mRNA to repress its translation and to the 3’ untranslated region of transferrin mRNA to prevent its degradation. Under iron-replete conditions, IRPs fail to bind, resulting in increased ferritin and decreased TfR1 levels [65].

We observed variable changes in levels of iron homeostatic proteins across cell lines and treatments. In senescent OVCAR3 cells, we observed increased IRP1, IRP2 and TfR1 levels. OVCAR4 cells showed dependence on the senescence inducer with cisplatin-induced senescent cells showing increased IRP1 and TfR1 levels and CX-5461-induced senescent cells showing decreased IRP1, IRP2 and TfR1 levels. OVCAR8 cells had low levels of TfR1 and showed an increased level of IRP2 in TIS. In spite of these maladaptive levels of iron homeostasis regulators, consistently across senescent OVCAR3, OVCAR4 and OVCAR8 cells independent of the senescence inducer, we observed a decreased level of the nuclear receptor coactivator NCOA4, the cargo receptor for turnover of ferritin via autophagy, termed ferritinophagy [66]. Collectively, these data highlight that iron overload in senescent cells likely results from impaired ferritinophagic turnover of iron stores along with aberrant regulators of iron homeostasis, thereby contributing to oxidative stress and ferroptotic priming. In support of this, iron chelation with deferoxamine alone was sufficient to promote senescent cell survival (Fig. S10A–D) and even resulted in the escape of OVCAR4 cells from CX-5461-induced senescence (Fig. S10B, D).

## Discussion

The increasing recognition that therapy-induced senescence is a reservoir for cancer recurrence underscores the critical need to identify senescent cancer cells and devise approaches for their eradication. We used transcriptome profiling to develop and validate a core gene expression signature, which will provide a valuable resource for future investigation of the therapy-induced senescent state in HGSOC. A focused screen for drugs causing cell death revealed a hitherto unappreciated vulnerability of senescent HGSOC cells to ferroptosis induction. Senescent HGSOC cells show dysregulated iron homeostasis contributing to iron overload and ferroptotic priming. Excess iron catalyses the formation of hydroxyl radicals via Fenton reactions, contributing to oxidative stress, which is tempered by an intrinsic antioxidant response. Our findings also warrant further investigation of elevated iron as a new TIS hallmark in HGSOC and the identification of novel mechanisms to target persistent cancer cells.

Despite our identification of markers indicative of a senescent-like phenotype in HGSOC, a major challenge in the investigation of senescence in cancer is the lack of universal markers to identify senescent cells. A previous report characterised TIS in breast, lung, colon and liver cancer cells and used machine learning to develop a senescence classifier [67]. Therefore, we reasoned that analysis of gene expression could provide another method to distinguish the TIS phenotype in HGSOC and facilitate identification of senescent HGSOC cells in vitro and in patients. We derived a core HGSOC_TIS signature and compared its predictive value with other published senescence signature using publicly available single-cell RNAseq data from patients prior to and following treatment with NACT. Although further evaluation is needed to confirm the predictive potential of the HGSOC_TIS signature, assessing its prognostic significance is important. This can be achieved by analysing sequencing data from post-treatment samples and correlating them with patient survival outcomes.

Our HGSOC_TIS signature highlights ROS as a key mediator of cellular senescence, operating at the crossroads of multiple signalling pathways, including TNF and NF-κB [68, 69]. Chemotherapy-induced DNA damage triggers stress-induced premature senescence, which is frequently accompanied by increased ROS production and accumulation [70]. ROS causes oxidative damage on macromolecules including nucleic acids, proteins and lipids, thereby amplifying the DNA damage response and leading to cell cycle arrest. ROS also impairs mitochondrial functions, initiating a feed-forward loop that further enhances ROS generation. Importantly, ROS can activate IκB kinase complexes, leading to phosphorylation and proteasomal degradation of IκBα, enabling nuclear translocation of NF-κB. Once in the nucleus, NF-κB functions as a master regulator of pro-inflammatory gene expression, driving the SASP program and reinforcing the senescence state [30, 71, 72].

While recent publications have shown a correlation between BRCA1 and ferroptosis sensitivity [73, 74], our data from a range of HGSOC cell lines with different genetic backgrounds suggest that in therapy-induced senescent cells, dysregulated iron homeostasis dictates sensitivity to ferroptosis. How exactly senescent cells accumulate iron remains an open question. Studies have shown that iron accumulation can result from increased expression of iron metabolism genes [60, 75, 76]. However, we did not observe an increase in iron metabolism genes at the transcriptional level, highlighting a yet undefined post-transcriptional mechanism leading to iron accumulation.

Our findings indicate that therapy-induced senescent cells are in a state of iron overload that is counterbalanced by protective mechanisms. Therapy-induced senescent cells exhibit high levels of both total and labile iron. Excess iron can induce oxidative stress [77], reinforcing senescence through DNA damage and mitochondrial dysfunction, creating a feed-forward loop of iron accumulation and reactive oxygen species production. To limit iron availability, senescent cells upregulate FTH1 to sequester excess iron. We also observed a low protein level of the selective ferritin cargo receptor NCOA4 in therapy-induced senescent cells, suggesting impaired ferritinophagy, which may hinder autophagic degradation of ferritin and the release of iron from ferritin. Meanwhile, therapy-induced senescent cells upregulate GPX4 and SOD2 to prevent the buildup of lipid peroxides and mitigate oxidative stress. However, these protective responses are inadequate to restore iron homeostasis or prevent iron-dependent lipid peroxidation as evident by the high labile iron pool in senescent cells, which favours ferroptosis by the introduction of additional stress, for example through GPX4 inhibitors, as demonstrated in our study.

Iron chelation strategies have been proposed to augment immune control in murine transplantable models of ovarian cancer [76, 78]. However, our findings suggest that caution may be warranted in the context of therapy-induced senescent human HGSOC, given that iron chelation had no effect on OVCAR3 and OVCAR8 cells, and promoted the intrinsic escape of OVCAR4 cells from CX-5461-induced senescence likely by mitigating the labile iron pool and oxidative stress. A recent report detailed the potential use of a labile iron pool-activated prodrug trioxolane coupled to a cytotoxic chemotherapeutic cyclopropylbenzidoline (TRX-CBI) [60]. To improve the effectiveness of this strategy, we hypothesise that such a prodrug could be modified to include a payload of a GPX4 inhibitor to induce ferroptosis in therapy-induced senescent cells.

Despite the traditional notion that senescent cells can resist apoptosis, we have demonstrated they remain susceptible to alternative forms of cell death such as ferroptosis, which could provide an alternative approach for their eradication to treat cancer following DNA-damaging therapy, particularly for HGSOC.

## Materials and methods

### Cell culture

Human HGSOC cell lines (OVCAR3, OVCAR4 and OVCAR8) were obtained from the National Cancer Institute. All cell lines were short tandem repeat (STR) profiled to confirm their authenticity and routinely tested for mycoplasma by PCR. AOCS14 and AOCS30 patient-derived lines were established from ascites drained from patients with HGSOC, as previously described [3]. Ethics approval was obtained for access to patient-derived cell lines generated by the Australian Ovarian Cancer Study (AOCS) (Peter MacCallum Cancer Centre HREC no. 15/84) and all methods were performed in accordance with the relevant guidelines and regulations. Written informed consent for their use in this study was obtained from all participants. Cells were cultured in RPMI 1640 medium (Thermo Fisher Scientific) containing 10% foetal bovine serum (Bovogen Biologicals, Keilor East, Australia), 1% GlutaMAX (Thermo Fisher Scientific) in a humidified 37 °C 5% CO_2_ incubator. Cisplatin (Accord Healthcare, Melbourne, Australia) in 0.9% saline was purchased from the Peter MacCallum Cancer Centre pharmacy. CX-5461 was purchased from SYNkinase (Parkville, Australia) and prepared in 50 mM NaH_2_PO_4_.

### Western blotting

Cells were washed twice with phosphate-buffered saline (PBS) and scraped into Western solubilisation buffer (0.5 mM EDTA, 20 mM HEPES, 2% SDS, pH 7.9). Samples were boiled at 95 °C for 10 min and sheared ten times using a 26-gauge needle. Protein concentration was determined using the detergent-compatible protein assay kit (Bio-Rad, Hercules, CA, USA #5000116) according to the manufacturer’s instructions. Protein was mixed with 6× sample loading buffer (375 mM Tris-HCl, 9% SDS, 50% Glycerol, 0.03% Bromophenol blue), re-boiled and stored at −20 °C. Equal amounts of protein (10–40 µg/lane) were loaded onto 4–15% Mini-PROTEAN TGX^TM^ Precast gels and separated by SDS-PAGE using Tris-glycine-SDS running buffer (25 mM Tris, 192 mM glycine, 0.1% SDS, pH 8.7) at 120 V for 75 min. Proteins were transferred to PVDF membranes in transfer buffer (25 mM Tris, 192 mM glycine, 20% methanol) at 250 mA for 45 min at 4 °C. Membranes were blocked in Tris-buffered saline containing 0.1% Tween 20 (TBST) and 5% non-fat milk for 1 h at room temperature. Membranes were incubated with primary antibodies at 4 °C overnight. After primary antibody incubation membranes were washed in TBST and incubated with HRP-conjugated secondary antibodies for 1–2 h at room temperature. Membranes were washed and imaged via chemiluminescence detection using Bio-Rad Clarity Western ECL substrate. Images were acquired using a ChemiDoc imaging System (Biorad #170-01401). Antibodies used a listed in Table S8. Images of full-length original Western blots are included in Supplementary File – Original Western blots.

### Cell cycle analysis

OVCAR3, OVCAR4, OVCAR8 cells were incubated with 10 µM BrdU (Sigma-Aldrich, St. Louis, MO, USA) for 30 min. AOCS14 and AOCS30 cells, given that they have a longer doubling time, were incubated with BrdU for three hours. Media was then collected, cells were washed with PBS and trypsinised. The PBS wash and trypsinised cells were collected into the same tube. One hundred µL of the cell suspension was used for cell counts. All samples were normalised to the same number of cells (1 × 10^6^ cells), then centrifuged at 400 × *g* for 5 min, and the supernatant was removed. Cells were resuspended in 1 mL PBS and fixed with 5 mL ice-cold 80% ethanol added dropwise whilst vortexing and incubated at 4°C overnight.

Fixed cells were centrifuged at 400 × *g* for 5 min, the supernatant was aspirated, and cells were resuspended in 2 N HCl containing 0.5% (v/v) Triton-X-100 for 30 min at room temperature. Cells were then centrifuged and resuspended in 0.1 M Na_2_B_4_O7·10H_2_O (pH 8.5) and incubated for ten minutes to neutralise the acid. Cells were then centrifuged and resuspended in 100 µL PBS + 2% FBS + 0.5% Tween 20 containing anti-BrdU antibody at concentration of 20 µL/mL and incubated for 30 min at room temperature. Cells were washed, centrifuged, and resuspended in 100uL PBS + 2% FBS + 0.5% Tween 20 containing FITC anti-mouse IgG antibody at a concentration of 10 µL/mL and incubated in the dark for 30 min. Cells were then washed, centrifuged, and resuspended in PBS + 2% FBS containing 10 µg/mL PI and 200 µg/mL RNase A, transferred to FACS tubes and incubated in the dark for 15 mins prior to analysis on a Canto II flow cytometer (BD Biosciences, Franklin Lakes, NJ, USA) where a minimum of 10,000 events were collected. Data analysis was performed using FlowJo.

### Flow cytometry of SA-β-gal activity

SA-β-gal activity was measured using a fluorescent dye, 9H-(1,3-dichloro-9,9-dimethylacridin-2-one-7-yl) β-D-galactopyranoside (DDAOG) (Thermo Fisher Scientific, Waltham, MA, USA, Cat #D6488), which is excited with a 633 nm laser and has an emission maximum at 660 nm. Cells were pre-treated with 100 nM bafilomycin A1 for 30 min to neutralise lysosomal pH. Cells were then lifted with Trypsin-EDTA, centrifuged at 400 × *g* for 4 min, and media were removed. Cells were either resuspended in media (unstained control) or media + 20 µM DDAOG for 1 h. Samples were then spun down, washed with PBS, resuspended in 2% FBS in PBS + propidium iodide (PI) and analysed on a flow cytometer. Single viable (PI negative) cells were analysed for DDAOG positivity.

### RNA sequencing and analysis

RNA isolation and purification were performed using the ISOLATE-II kit (Bioline, London, United Kingdom #52073) according to the manufacturer’s instructions. RNA was eluted in RNase-free H_2_O and quantified using a NanoDrop ND-1000 spectrophotometer. RNA integrity was then assessed on an Agilent Tapestation. Library preparation and 3’ RNA-sequencing and were performed by the Molecular Genomics Core at the Peter MacCallum Cancer Centre using the Illumina NextSeq 2000 platform (single-end 100 bp).

The raw sequencing reads were aligned to human (HG19) reference genome using HISAT2 (v2.0.4), and gene expression was quantified using featurecount (v2.0.3) software. Normalised expression was measured in count-per-million (CPM) in log2 scale, with library size adjustment using Trimmed Mean of M-values method [79]. Differential expression analysis was performed following LIMMA-Voom workflow [80]. Heatmaps were generated using ComplexHeatmap R package to visualise normalised expression data, using Pearson correlation distance to compare across genes and Euclidean distance to compare across samples. The colour scale of each gene is centered at the average normalised expression across samples in the heatmap. Gene set enrichment analysis was performed using the FGSEA R package [81] to calculate the enrichment level of a given gene set through Normalised Enrichment Score (NES) metrics, where the significance is evaluated through permutation testing. Bubble plots, scatter plots were generated using ggplot2 R package.

### Drug screen

Three million OVCAR8 cells stably expressing H2B-GFP, generated by lentiviral transduction according to methods previously described [82] using LV-GFP, a gift from Elaine Fuchs (Addgene plasmid #25999), were seeded into T175 flasks. The following day, cells were treated with senescence-inducing drugs (cisplatin at 10 µM or CX-5461 at 3 µM). At 48 h post drug treatment, cells were lifted with PBS + 10 mM EDTA, counted and seeded at 5 × 10^3^ cells/well (cisplatin treatment) or 3.5 × 10^3^ cells/well (CX-5461 treatment) into PhenoPlate 384-well plates (Revvity, Waltham, MA, USA #6057328) using an EL406 dispenser (Agilent, Santa Clara, CA, USA). At 6 d post seeding into 384-well plates, media was replaced with fresh RPMI complete media using the EL406. Control compounds and Incucyte Cytotox Red Dye (Sartorius, Göttingen, Germany #4632) (1:8000) were dispensed using a D300e Digital Dispenser (Tecan, Männedorf, Switzerland). Compound source plates (1131 compounds, Compounds Australia, Griffith University, Australia) containing dried compounds were hydrated with media using a Janus G3 robot (Revvity) and transferred to cell plates for a final concentration of at 1 µM and 5 µM (transferred at 1:10 dilution). The plates were then imaged using an Incucyte SX5 live cell imager every four hours for 24 h. Staurosporine (#S1421) Mitomycin C (#S8146) were purchased from Selleckchem (Houston, TX, USA). The following drugs were purchased from MedChemExpress (Monmouth Junction, NJ, USA): ML-210 (#HY-100003), RSL3 (#HY-100218A), FIN56 (#HY-103087), N6F11 (#HY-162065), ABT-263 (#HY-10087), ABT-737 (#HY-50907), A-1155463 (#HY-19725), Erastin (#HY-15763), CIL-56 (#HY-112063). All drugs were reconstituted in DMSO.

Controls compounds used were: ABT-263 (50, 20, 10, 5, 3, 1, and 0.1 µM), ABT-737 (50, 20, 10, 5, 1, and 0.1 µM), A-1155463 (50, 20, 10, 5, 1 and 0.1 µM), Mitomycin (100, 10, 1 and 0.1 µM) and Staurosporine (50, 10, 1 and 0.1 µM). At 24 h post-compound treatment, cells were fixed with 4% paraformaldehyde and stained with 2 µg/mL DAPI and 0.4 U/mL rhodamine-phalloidin (Biotium #00027) diluted in PBS + 0.3% Triton X-100. Plates were imaged on the CellInsight CX7 LZR platform (Thermo Fisher Scientific) at 10X magnification using the blue (405 nm) and red (561 nm) excitation lasers and BGRFRN emission filters. Images were acquired at 10X magnification for nine fields per well (~80% coverage of the total well area) and analysed for cell counts using a custom pipeline generated in CellProfiler (version 4.1.3).

Cell nuclei were identified in the DAPI channel using the Otsu thresholding method with an adaptive thresholding strategy. Illumination correction and Median Filter smoothing were applied to the rhodamine-phalloidin channel enhance cell boundaries, which were identified using the Minimum Cross-Entropy method with a global thresholding strategy. The segmentation settings were optimised for each cell line. Dead cells that were not washed away during fixing and staining were identified by their lack of cytoplasm staining in the rhodamine-phalloidin channel and excluded from analysis. The per-image field cell counts were aggregated into a final total cell count for each well. The per-well cell counts were normalised for potential batch effects by fold changing to the median cell count of the vehicle control (DMSO) wells on the same plate. Robust Z-scores were then calculated from the mean fold change values for each compound (averaged across two replicate plates) using the following formula:

Robust *Z*-score = (value − median of all sample values)/median absolute deviation of all sample values

Hit compounds were identified as resulting in a robust Z-score ≤ −3 in both cisplatin and CX-5461 senescent cells at either concentration tested.

### FerroOrange and C11-BODIPY staining

Cells were lifted with Trypsin-EDTA, neutralised with media, and washed once with PBS and transferred to a well of a V-bottom plate. Cells were resuspended in serum-free media containing 1 µM FerroOrange (Cell Signaling Technology, Danvers, MA, USA #36104) and 1:1000 LIVE/DEAD Fixable Near-IR (780) Stain (Thermo Fisher Scientific #L34994). Cells were incubated at 37°C 5% CO_2_ for 30 min and resuspended in PBS containing 1 µM FerroOrange and 1:1000 LIVE/DEAD Fixable Near-IR Stain. Single viable (Near-IR negative) cells were analysed on a Canto II flow cytometer (BD Biosciences) and median fluorescence intensity for FerroOrange (excitation/emission maxima 543 nm/580 nm) or C11-BODIPY (excitation/emission maxima 488 nm/510 nm after oxidation) was quantified.

### DCFHDA and MitoSOX staining and mitochondrial inner membrane potential detection

2′,7′-dichlorofluorescein diacetate (DCFHDA, 10 µM; Sigma-Aldrich, St. Louis, MO, USA #35845) and MitoSOX Red mitochondrial superoxide indicator (5 µM, Invitrogen, Waltham, MA #M36008) were added to cell culture media and incubated at 37°C 5% CO_2_ for 1 h.

The membrane potential was measured using mitochondrial membrane potential assay kit (Cell Signaling Technology, Danvers, MA, USA #13296). Tetramethylrhodamine (TMRE) (Invitrogen, Waltham, MA #T669) at 0.2 µM was added to cell culture media and incubated at 37 °C 5% CO_2_ for 1 h.

After staining, cells were trypsinised and resuspended in 200 µL PBS containing 0.5 µg/mL DAPI. Cells were analysed on a BD LSR Fortessa flow cytometer and median fluorescence intensity for DCFHDA (excitation/emission maxima 504 nm/524 nm), MitoSOX Red indicator (excitation/emission maxima 396 nm/610 nm) and TMRE (excitation/emission maxima 550 nm/575 nm) in live cells were quantitated. The live cells were determined by assessing DAPI-negative populations. Data were analyzed using FlowJo software (Version 10.10.0).

### Label-free proteomics and mass spectrometry

Cells were washed twice with PBS and harvested with 100 µL guanidinium chloride (GdnCl) lysis buffer per sample (6 M GdnCl, 100 mM Tris pH 8.5, 10 mM TCEP, 40 mM chloroacetamide). Samples were heated at 95 °C for 10 min and stored at −80 °C prior to analysis. Four different biological replicates were collected per cell line per treatment. Samples were thawed and protein was quantified using a BCA Protein Assay Kit (Pierce, Waltham, MA, USA #23227). One hundred µg of protein in a 200 µL volume was precipitated with 1 mL acetone and 300 µL methanol overnight at −20 °C. The protein precipitates were washed twice with 80% acetone and resuspended in 50 mM Tris-HCl pH 8. Twenty micrograms protein was digested by Trypsin/Lys-C Mix, Mass Spec Grade (Promega, Madison, WI, USA #V5071) at 1:50 enzyme-to-protein ratio at 37 °C, 1000 rpm overnight. Samples were then acidified with 1.0% (v/v) formic acid to terminate digestion, desalted with Oasis HLB SPE cartridges (Oasis HLB 1cc Vac Cartridge, 10 mg) (Waters Corporation, Milford, MA, USA #186000383) and eluted with 60% acetonitrile and 5% formic acid. After speed vacuum evaporation, proteins were resuspended in 2% acetonitrile and 0.05% trifluoroacetic acid (TFA) for LC-MS analysis in the Bio21 Mass Spectrometry and Proteomics Facility at the University of Melbourne. Peptide mixtures were analysed by nanoLC-MS/MS suing the Q Exactive Plus Orbitrap Mass Spectrometer (Thermo Fisher Scientific).

### Protein identification and analysis

Raw data was processed using MaxQuant with the Andromeda search engine for protein and peptide identification. The results were searched against a Homo Sapiens database using the default search parameters. Enzyme specificity was Trypsin/P with a maximum of two missed cleavages; fixed modifications of Carbamidomethyl (C) and variable modifications of Oxidation (M); Acetyl (Protein N-term). Proteins were quantified using the LFQ value from MaxQuant using largely the default settings with the additional parameter of match between runs also being selected. Proteins with ≥ 2 peptides identified in ≥ 2 replicates were included in the analysis.

The processed data were analysed with the MSstats package (4.6.5) via RStudio (version 4.2.0). Raw data were cleaned (reverse & contaminants removed) and features with less than 3 measurements across runs were removed. Cleaned data were then processed with the dataProcess function, using the TMP summary method, log2 transformed, normalised based on median, and summarisation was performed. Group comparisons were done comparing each senescent treated group to its equivalent control group. This tested for differentially abundant proteins with models, resulting in an output of all identified proteins and their log2FC and statistical analyses between the comparisons chosen. Significantly differentially expressed proteins were considered those with a fold change FC+/−1.5 and adjusted p-value of <0.05.

### Inductively coupled plasma mass spectrometry (ICP-MS)

Cell pellets containing 2 × 10^6^ cells were lysed in 30 µL 65% nitric acid (HNO3) (Suprapure Nitric Acid Supelco). Samples were heated at 90 °C for 20 min and allowed to cool. Samples were diluted with ultrapure water (18.2 MΩ; Merck Millipore, Australia) to 2% HNO_3_ final concentration. Samples were centrifuged at 15,000 × *g* for 15 min and supernatant was transferred to a new tube. Sample blanks were prepared identically as samples for analysis.

An Agilent 8900 triple quadrupole ICP-MS (Agilent Technologies) was tuned and optimised using a tuning solution containing 1 μg/L of cerium (Ce), cobalt (Co), lithium (Li), thallium (Tl) and Y in 2% (v/v) HNO3 (Agilent Technologies, Australia). The ion intensity at m/z 56 (Iron) was monitored in a helium gas analysis mode. The instrument was calibrated using a 12-point calibration curve for Iron using commercially available multi-element standards at 0, 0.5, 1, 2.5, 5, 10, 25, 50, 100, 250, 500 and 1000 parts per billion (ppb) in 1% HNO3 (Multi-Element Calibration Standard 2 A, Agilent Technologies, USA). Yttrium (89Y), Scandium (45Sc), and Indium (115In) (Agilent Technologies) were used as an internal reference elemental standard at a concentration of 0.1 μg/mL and used to normalise recovery across all samples. All samples, calibration standards and internal standards were introduced to the nebuliser using a peristaltic pump and T-piece for sample mixing at the flow rate of 0.4 mL/min. The sample uptake time was 25 s and the stabilisation time was 15 s. The data was collected in spectrum mode with the average of three technical replicates, 50 sweeps and 3 points across the peak. The ICP-MS operating parameters were established following the manufacturer’s guidelines, with further optimisation tailored specifically for iron analysis in a batch-specific mode prior to each experiment. The instrument was operated in Single Quad scan type with an RF power of 1550 W and an RF matching voltage of 1.2 V. The nebulizer gas flow rate was maintained at 1.12 L/min, while the extraction voltages were set at −12 V for Extract 1 and -220 V for Extract 2. The Omega Bias and Omega Lens voltages were optimized to −125 V and 7.4 V, respectively, with the Deflect voltage adjusted to -3.8 V. Helium gas was introduced at a flow rate of 4.3 mL/min to enhance sensitivity and reduce interference. The Octopole Bias was maintained at −18 V to ensure optimal performance during iron analysis. The Limit of Detection (LOD-lowest analyte concentration distinguishable from the blank) and Limit of Quantification (LOQ-lowest concentration quantifiable with acceptable accuracy and precision) were determined to evaluate the sensitivity and reliability of the analytical method. The calculations were performed using the Standard Deviation of Blank Method. For the sample blank, the LOD was calculated as 2.8955 µg/L, and the LOQ as 5.5352 µg/L. The Blank Equivalent Concentration (BEC) was 1.9874 µg/L, indicating that both the LOD and LOQ exceed the baseline signal of the blank. This ensures that the method is suitable for detecting and quantifying analyte concentrations above the baseline noise. Furthermore, all sample concentrations were significantly higher than both the LOD and LOQ, confirming the method’s sensitivity, accuracy, and precision for the dataset.

### Multiplex Immunohistochemistry of FTH1 and WT1

Samples from the AOCS cohort, collected at primary surgery, recurrence, and research autopsy timepoints, were used for fluorescent multiplex IHC on tumour microarrays (TMAs) representing 14 patients. The TMAs consisted of two cores per sample, resulting in a total of over 900 cores. Staining with FTH1, coupled with WT1, was used to visualise the accumulation of FTH1 within the tumour cells.

TMA blocks were sectioned at 4μm and mounted Superfrost Plus glass slides (Thermo Scientific, Eugene, OR, USA)), dried for at least 12 h, and then underwent the manufacturer’s dewaxing protocol in the Leica autostainer XL (Leica Biosystems, Wetzlar, Germany). Antigen retrieval was performed in a pressure cooker at 125 °C for 3 min. Slides were then stained using the primary antibodies listed in Table S8 according to manufacturer’s benchtop protocol using the Opal 6-Plex Manual Detection Kit (Akoya Biosciences, Marlborough, MA, USA).

Multispectral component data images were taken using the Vectra 3 microscope system and Phenochart (PerkinElmer, Waltham, MA, USA). Composite images for each slide were constructed from component data images using inForm Viewer. Final images were analysed using the image analysis platform, HALO (v3.5, Indica Labs, Albuquerque, NM, USA) using the Highplex FL module.

### Statistical analysis

All details related to the sample sizes are detailed in the Figure Legends. The sample size was chosen based on common practice and previous experience. The sample size was not determined based on power calculations. However, we consistently achieved at least three biological replicates for all experiments. All experimental data shown in plots are represented as mean ± SD/SEM, and the exact number of independent replicates for each experiment is stated in figure legends. The statistical methods and their justification are described in the “Material and methods” section or figure legends.

## Supplementary information

Supplementary File - AOCS Study Group and Affiliations

Supplementary Figures

Original Western Blots

Supplementary Tables

## Data Availability

Signature validation analysis on public single cell RNASeq (scRNAseq) datasets [38, 43] was performed using Seurat workflow [83] in conjunction with the pseudobulk analysis method, which aggregates abundance per-sample and then applies bulk RNAseq analysis methods such as DE analysis using the aforementioned LIMMA-Voom workflow. FastQ raw data and processed files are available in the public depository NCBI GEO under accession number GSE286888.
