# Supplementary material for: Exploiting dysregulated iron homeostasis to eradicate persistent high-grade serous ovarian cancer
Source: Cell Death Discov. 2025 Sep 25;11:423. doi: 10.1038/s41420-025-02716-1 (PMC12462457; doi:10.1038/s41420-025-02716-1)
Supplement: Supplementary file 1 — Supplementary File - AOCS Study Group and Affiliations [file 41420_2025_2716_MOESM1_ESM.docx]

***AOCS STUDY GROUP***

***Management Group****:* D Bowtell^1,3,4,5,6^, G Chenevix-Trench^2^, P Webb^2^, A DeFazio^7,8,9^, E Christie^1,4^, D Garsed^1,4^, A Green^2^, D Gertig^10^

***Project and Data Managers****:* N Traficante^1,4^, S Fereday^1,4^, S Moore^2^, J Hung^7^, K Harrap^2^, T Sadkowsky^2^, N Pandeya^2^

***Research Nurses and Assistants***:

M Malt^2^, A Mellon^11^, R Robertson^11^, T Vanden Bergh^12^, M Jones^12^, P Mackenzie^12^, J Maidens^13^, K Nattress^14^, YE Chiew^7^, A Stenlake^9^, H Sullivan^9^, B Alexander^2^, P Ashover^2^, S Brown^2^, T Corrish^2^, L Green^2^, L Jackman^2^, K Ferguson^2^, K Martin^2^, A Martyn^2^, B Ranieri^2^, J White^15^, V Jayde^16^, P Mamers^17^, L Bowes^1^, L Galletta^1^, D Giles^1^, J Hendley^1^, K Alsop^1^, T Schmidt^18^, H Shirley^18^, C Ball^19^, C Young^19^, S Viduka^18^, Hoa Tran^18^, Sanela Bilic^18^, Lydia Glavinas^18^, Julia Brooks^20^

***Clinical and Scientific Collaborators****:*

R Stuart-Harris^21^, F Kirsten^22^, J Rutovitz^23^, P Clingan^24^, A Glasgow^24^, A Proietto^11^, S Braye^11^, G Otton^11^, J Shannon^25^, T Bonaventura^26^, J Stewart^26^, S Begbie^27^ M Friedlander^28^ D Bell^13^, S Baron-Hay^13^, A Ferrier^13^ (*dec.*), G Gard^13^, D Nevell^13^, N Pavlakis^13^, S Valmadre^13^, B Young^13^, C Camaris^12^, R Crouch^12^, L Edwards^12^, N Hacker^12^, D Marsden^12^, G Robertson^12^, P Beale^14^, J Beith^14^, J Carter^14^, C Dalrymple^14^, R Houghton^14^, P Russell^14^, M Links^29^, J Grygiel^30^, J Hill^31^, A Brand^8,32^, K Byth^32^, R Jaworski^33^, P Harnett^8,32^, R Sharma^8,33^, G Wain^32^, B Ward^34^, D Papadimos^34^, A Crandon^35^, M Cummings^35^, K Horwood^35^, A Obermair^35^, L Perrin^35^, D Wyld^35^, J Nicklin^35,36^, M Davy^15^, MK Oehler^15^, C Hall^15^, T Dodd^15^, T Healy^37^, K Pittman^37,^ D Henderson^37^, J Miller^39^, J Pierdes^39^, P Blomfield^16^, D Challis^16^, R McIntosh^16^, A Parker^16^, B Brown^40^, R Rome^40^, D Allen^41^, P Grant^41^, S Hyde^41^, R Laurie^41^, M Robbie^41,^ D Healy^17^, T Jobling^17^, T Manolitsas^17^, J McNealage^17^, P Rogers^17^, B Susil^17^, E Sumithran^17^, I Simpson^17^, L Mileshkin^1^, G Au-Yeung^1^, K Phillips^1^, D Rischin^1^, S Fox^1^, D Johnson^1^, S Lade^1^, M Loughrey^1^, N O’Callaghan^1^, W Murray^1^, P Waring^3^, V Billson^42^, J Pyman^42^, D Neesham^42^, M Quinn^42^, C Underhill^43^, R Bell^44^, LF Ng^45^, R Blum^46^, V Ganju^47^, I Hammond^19^, Y Leung^19^, A McCartney^19^ (*dec.*), M Buck^48,^ I Haviv^49^, D Purdie^2^, D Whiteman^2^, N Zeps^18^

^1^Peter MacCallum Cancer Centre, Melbourne, Victoria, 3000, Australia.

^2^QIMR Berghofer Medical Research Institute, Brisbane, Queensland, 4006, Australia.

^3^Department of Pathology, University of Melbourne, Parkville, Victoria, 3052, Australia.

^4^Sir Peter MacCallum Cancer Centre Department of Oncology, University of Melbourne, Parkville, Victoria, 3052, Australia.

^5^Department of Biochemistry and Molecular Biology, University of Melbourne, Parkville, Victoria, 3052, Australia.

^6^Ovarian Cancer Action Research Centre, Department of Surgery and Cancer, Imperial College London, London, England, W12 0HS, UK.

^7^Centre for Cancer Research, The Westmead Institute for Medical Research, Sydney, New South Wales, 2145, Australia

^8^The University of Sydney, Sydney, New South Wales, 2006, Australia.

^9^Department of Gynaecological Oncology, Westmead Hospital, Sydney, New South Wales, 2145, Australia.

^10^Melbourne School of Population and Global Health, University of Melbourne, Parkville, Victoria, 3052, Australia.

^11^John Hunter Hospital, Lookout Road, New Lambton, New South Wales, 2305, Australia

^12^Royal Hospital for Women, Barker Street, Randwick, New South Wales, 2031, Australia

^13^Royal North Shore Hospital, Reserve Road, St Leonards, New South Wales, 2065, Australia

^14^Royal Prince Alfred Hospital, Missenden Road, Camperdown, New South Wales, 2050, Australia

^15^Royal Adelaide Hospital, North Terrace, Adelaide, South Australia, 5000, Australia

^16^Royal Hobart Hospital, 48 Liverpool St, Hobart, Tasmania, 7000, Australia

^17^ Monash Medical Centre, 246 Clayton Rd, Clayton, Victoria, 3168, Australia

^18^Western Australian Research Tissue Network (WARTN), St John of God Pathology, 23 Walters Drive, Osborne Park, Western Australia, 6017, Australia

^19^Women and Infant's Research Foundation, King Edward Memorial Hospital, 374 Bagot Road, Subiaco, Western Australia, 6008, Australia

^20^St John of God Hospital, 12 Salvado Rd, Subiaco, Western Australia, 6008, Australia

^21^Canberra Hospital, Yamba Drive, Garran, Australian Capitol Territory, 2605, Australia

^22^Bankstown Cancer Centre, Bankstown Hospital, 70 Eldridge Road, Bankstown, New South Wales, 2200, Australia

^23^Northern Haematology & Oncology Group, Integrated Cancer Centre, 185 Fox Valley Road, Wahroonga, New South Wales, 2076, Australia

^24^Illawarra Shoalhaven Local Health District, Wollongong Hospital, Level 4 Lawson House, Wollongong, New South Wales, 2500, Australia

^25^Nepean Hospital, Derby Street, Kingswood, New South Wales, 2747, Australia

^26^Newcastle Mater Misericordiae Hospital, Edith Street, Waratah, New South Wales, 2298, Australia

^27^Port Macquarie Base Hospital, Wrights Road, Port Macquarie, New South Wales, 2444, Australia

^28^Prince of Wales Clinical School, University of New South Wales, New South Wales, 2031, Australia

^29^St George Hospital, Gray Street, Kogarah, New South Wales, 2217, Australia

^30^St Vincent’s Hospital, 390 Victoria Street, Darlinghurst, New South Wales, 2010, Australia

^31^Wagga Wagga Base Hospital, Docker St, Wagga Wagga, New South Wales, 2650, Australia

^32^Crown Princess Mary Cancer Centre, Westmead Hospital, Westmead, Sydney, New South Wales, 2145, Australia.

^33^Department of Pathology, Westmead Clinical School, Westmead Hospital, The University of Sydney, New South Wales, 2006, Australia

^34^Mater Misericordiae Hospital, Raymond Terrace, South Brisbane, Queensland, 4101, Australia

^35^The Royal Brisbane and Women’s Hospital, Butterfield Street, Herston, Queensland, 4006, Australia

^36^ Wesley Hospital, 451 Coronation Drive, Auchenflower, Queensland, 4066, Australia

^37^Burnside Hospital, 120 Kensington Road, Toorak Gardens, South Australia, 5065, Australia

^38^Flinders Medical Centre, Flinders Drive, Bedford Park, South Australia, 5042, Australia

^39^Queen Elizabeth Hospital, 28 Woodville Road, Woodville South, South Australia, 5011, Australia

^40^Freemasons Hospital, 20 Victoria Parade, East Melbourne, Victoria, 3002, Australia

^41^Mercy Hospital for Women, 163 Studley Road, Heidelberg, Victoria, 3084, Australia

^42^The Royal Women’s Hospital, Parkville, Victoria, 3052, Australia

^43^Border Medical Oncology, Wodonga, Victoria, 3690, Australia

^44^Andrew Love Cancer Centre, 70 Swanston Street, Geelong, Victoria, 3220, Australia

^45^Ballarat Base Hospital, Drummond Street North, Ballarat, Victoria, 3350, Australia

^46^Bendigo Health Care Group, 62 Lucan Street, Bendigo, Victoria, 3550, Australia

^47^Peninsula Health, 2 Hastings Road, Frankston, Victoria, 3199, Australia

^48^Mount Hospital, 150 Mounts Bay Road, Perth, Western Australia 6000, Australia

^49^Faculty of Medicine, Bar-Ilan University, 8 Henrietta Szold St, Safed, Israel
