# Supplementary material for: Exploiting dysregulated iron homeostasis to eradicate persistent high-grade serous ovarian cancer
Source: Cell Death Discov. 2025 Sep 25;11:423. doi: 10.1038/s41420-025-02716-1 (PMC12462457; doi:10.1038/s41420-025-02716-1)
Supplement: Supplementary file 2 — Supplementary Figures [file 41420_2025_2716_MOESM2_ESM.pdf]

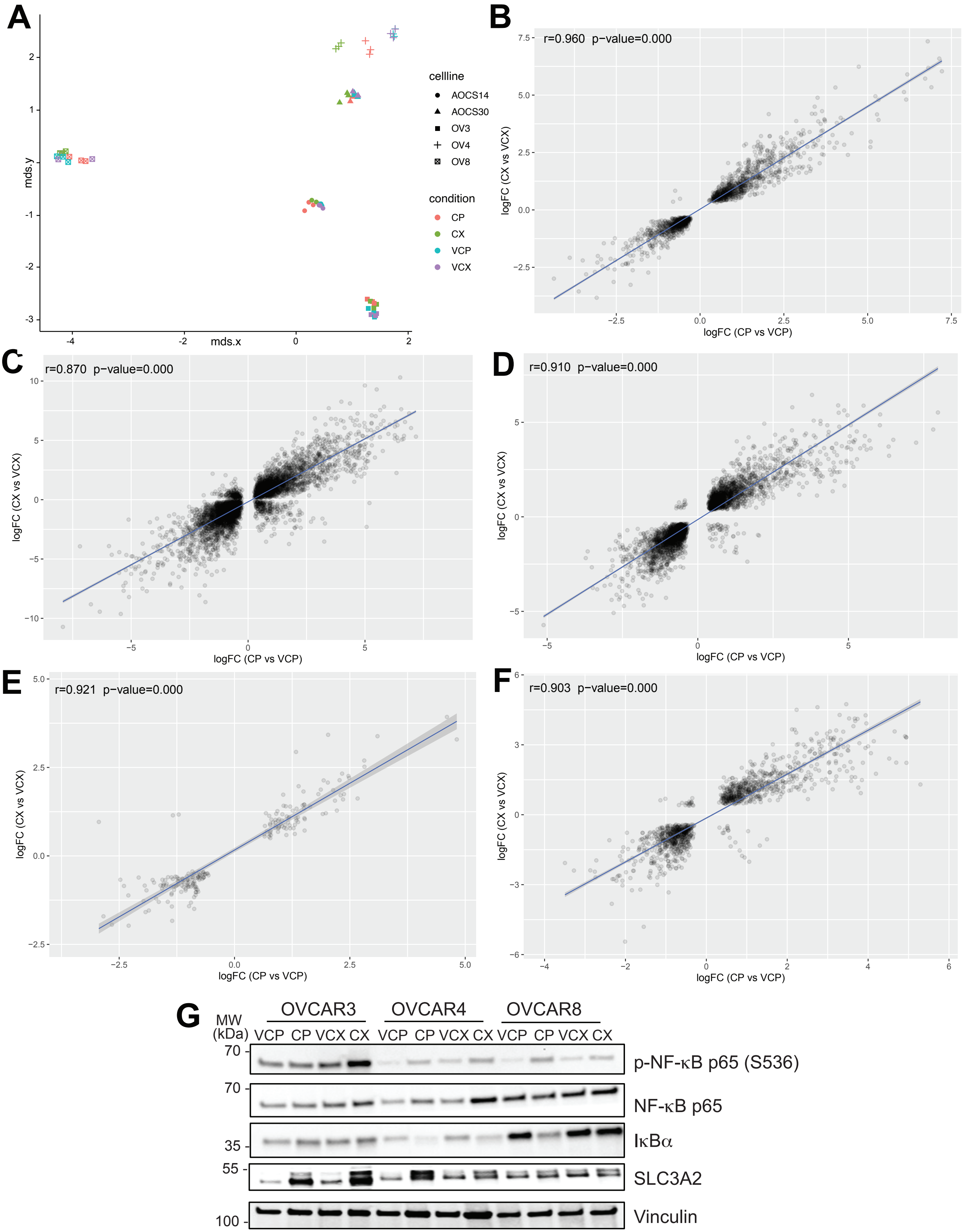

Figure S1. Therapy-induced senescent gene expression depends on lineage but not on senescence inducer. **(A)** MDS plot of RNAseq data for OVCAR3, OVCAR4, OVCAR8, AOCS14 and AOCS30 cells exposed to cisplatin (CP) or CX-5461 (CX) and their respective vehicle controls (VCP or VCX). **(B-F)** Correlation plots of differentially expressed genes for cisplatin or CX-5461 and their respective vehicles for **(B)** OVCAR3, **(C)** OVCAR4, **(D)** OVCAR8, **(E)** AOCS14 and **(F)** AOCS30 cells. **(G)** Representative Western blots of NF-κB signalling and SLC3A2 in OVCAR3, OVCAR4 and OVCAR8 cells exposed to cisplatin or CX-5461 and the vehicle controls. Vinculin was probed as a loading control.

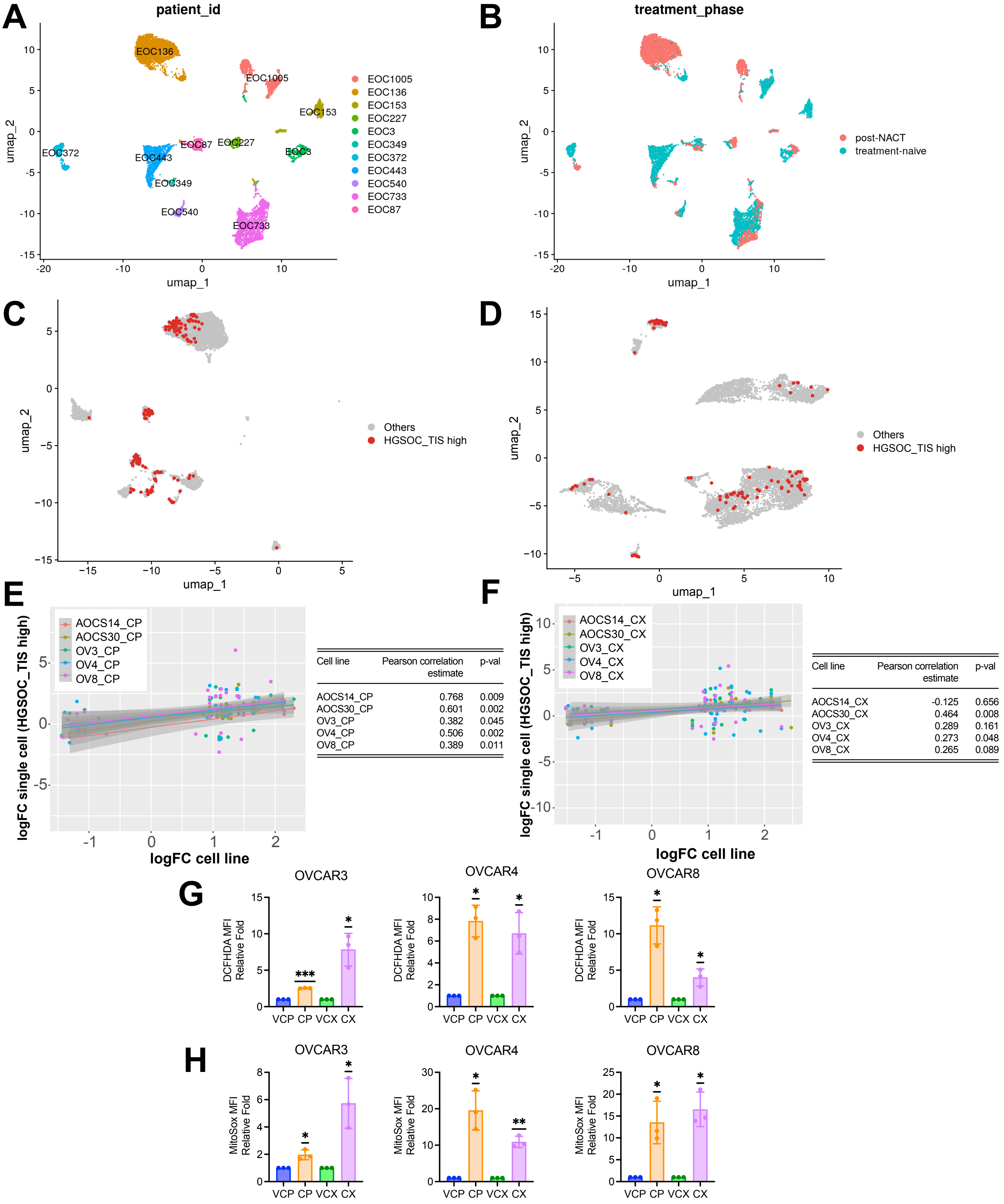

Cell line	Pearson correlation estimate	p-val
AOCS14_CP	0.768	0.009
AOCS30_CP	0.601	0.002
OV3_CP	0.382	0.045
OV4_CP	0.506	0.002
OV8_CP	0.389	0.011

Cell line	Pearson correlation estimate	p-val
AOCS14_CX	-0.125	0.656
AOCS30_CX	0.464	0.008
OV3_CX	0.289	0.161
OV4_CX	0.273	0.048
OV8_CX	0.265	0.089

Figure S2. Validation of core HGSOC TIS gene expression signature in external single cell RNAseq datasets. (A and B) UMAP of single cell RNAseq data from GSE165897 for **(A)** 11 patients with HGSOC and **(B)** paired treatment-naïve and post-NACT tumour samples. **(C and D)** UMAP of single cell RNAseq data showing post-treatment tumour cells with a high HGSOC_TIS signature expression score **(C)** GSE165897 or **(D)** GSE154600. **(E and F)** Correlation plot of differentially expressed genes from OVCAR3, OVCAR4, OVCAR8, AOCS14 and AOCS30 cellsexposed to **(E)** cisplatin or **(F)** CX-5461 and post-NACT single cell RNAseq samples (GSE154600) with a HGSOC_TIS high score > two median absolute deviations. Tables indicate Pearson correlation estimate values and their corresponding p-values for statistical significance.**(G and H)** Relative cytoplasmic and mitochondrial ROS levels as measured by **(G)** DCFHDA, iand **(H)** MitoSOX median fluoresence intensity (MFI), respectively, in CX-5461 or cisplatin treated cells compared to their respective vehicle controls. Data are presented as mean ± SEM from n = 3 independent experiment. Statistical analysis was performed using one sample t test. *, p < 0.05; **, p < 0.01; ***, p < 0.001.

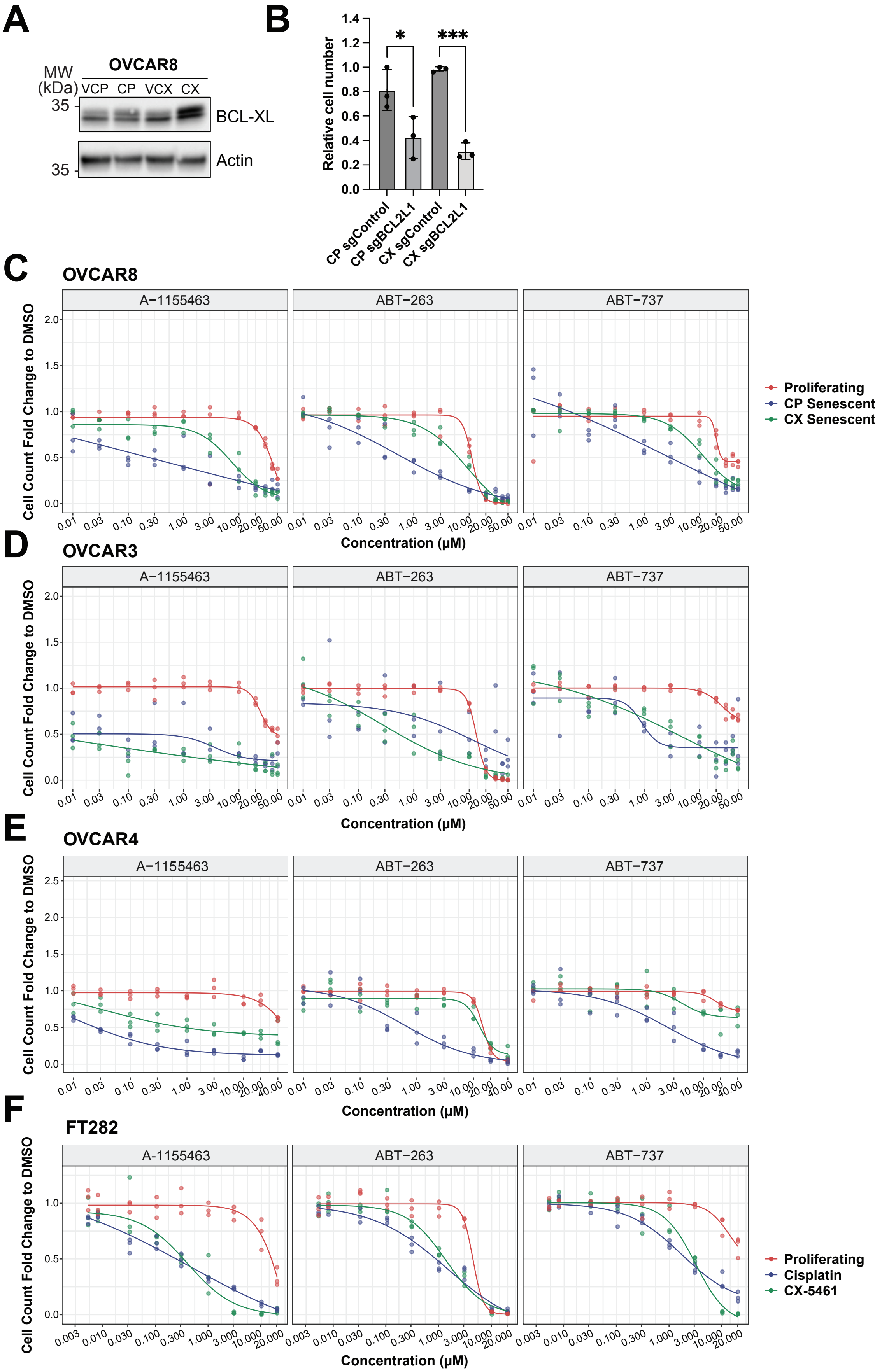

Figure S3. Therapy-induced senescent HGSOC cells exhibit varied responses to BCL-XL inhibitors. (A) Representative Western blot of BCL-XL expression in OVCAR8 cells made senescent by cisplatin (CP) or CX-5461 (CX) as compared to proliferating vehicle controls. Actin was probed as a loading control. **(B)** Quantification of relative cell number for therapy-induced senescent OVCAR8 Cas9 expressing cells transfected with control or BCL2L1 sgRNAs is shown as mean ± SEM from n=3 independent experiments. Statistical analysis was performed using one-way ANOVA with a Šídák's multiple comparisons test. *, $p < 0.05$; ***, $p < 0.001$. **(C-F)** Dose response curves of proliferating or therapy-induced senescent HGSOC **(C)** OVCAR8, **(D)** OVCAR3, **(E)** OVCAR4 and **(F)** fallopian tube epithelial FT282 cells exposed to increasing concentrations of BCL-XL inhibitors A-1155463, ABT-263 or ABT-737 for 24 h.

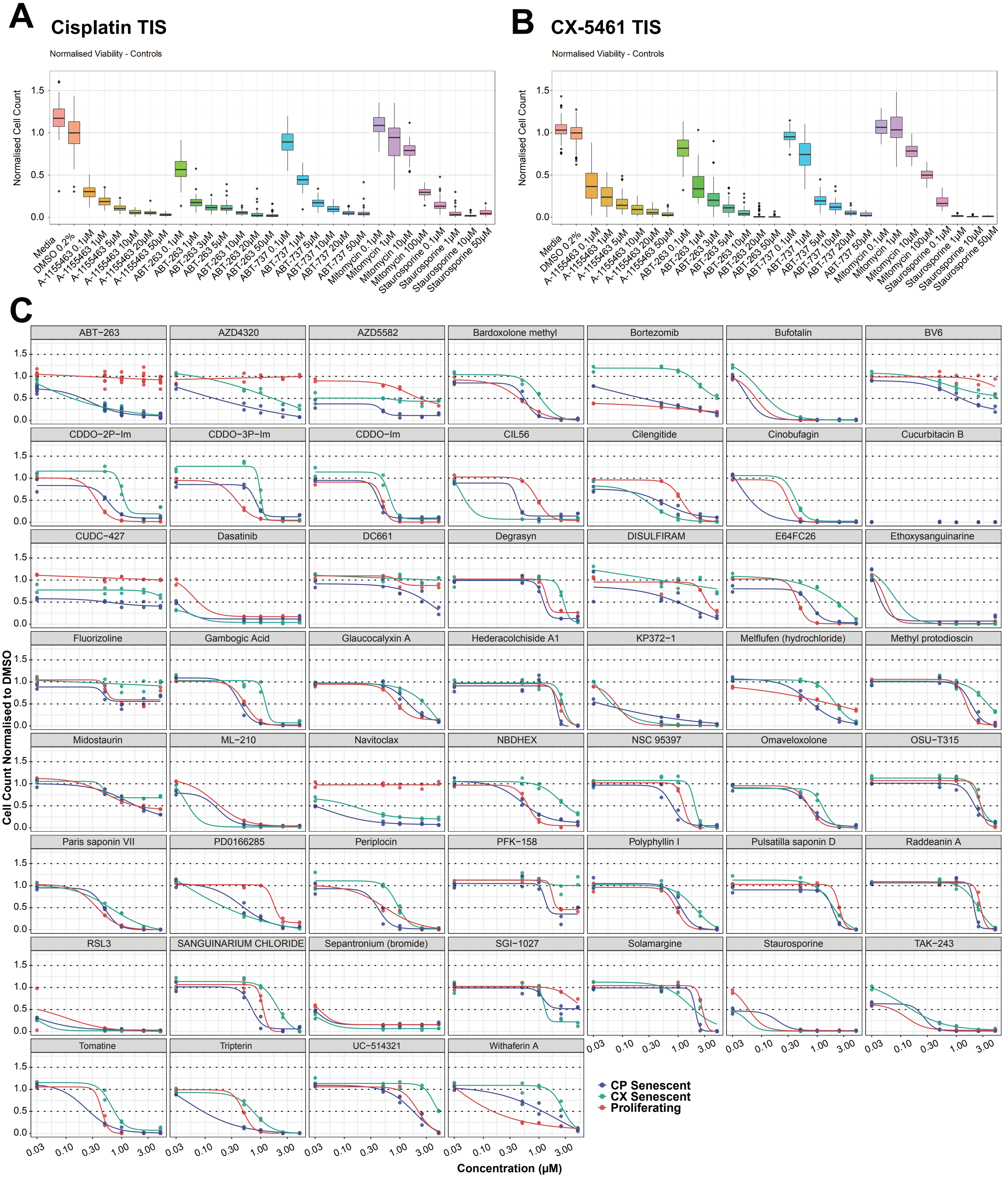

Figure S4. A drug screen identifies candidate eradicators of therapy-induced senescent HGSOC cells. (A and B) Quality control metrics for controls used in primary screen of OVCAR8 H2B-GFP cells made senescent by **(A)** cisplatin or **(B)** CX-5461 are shown as mean ± SD from n=6 wells for OVCAR8 H2B-GFP cells. **(C)** Dose response curves of OVCAR8 proliferating or senescent cells exposed to ABT-263 (positive control) and 52 compounds with Z-score ≤ -3.

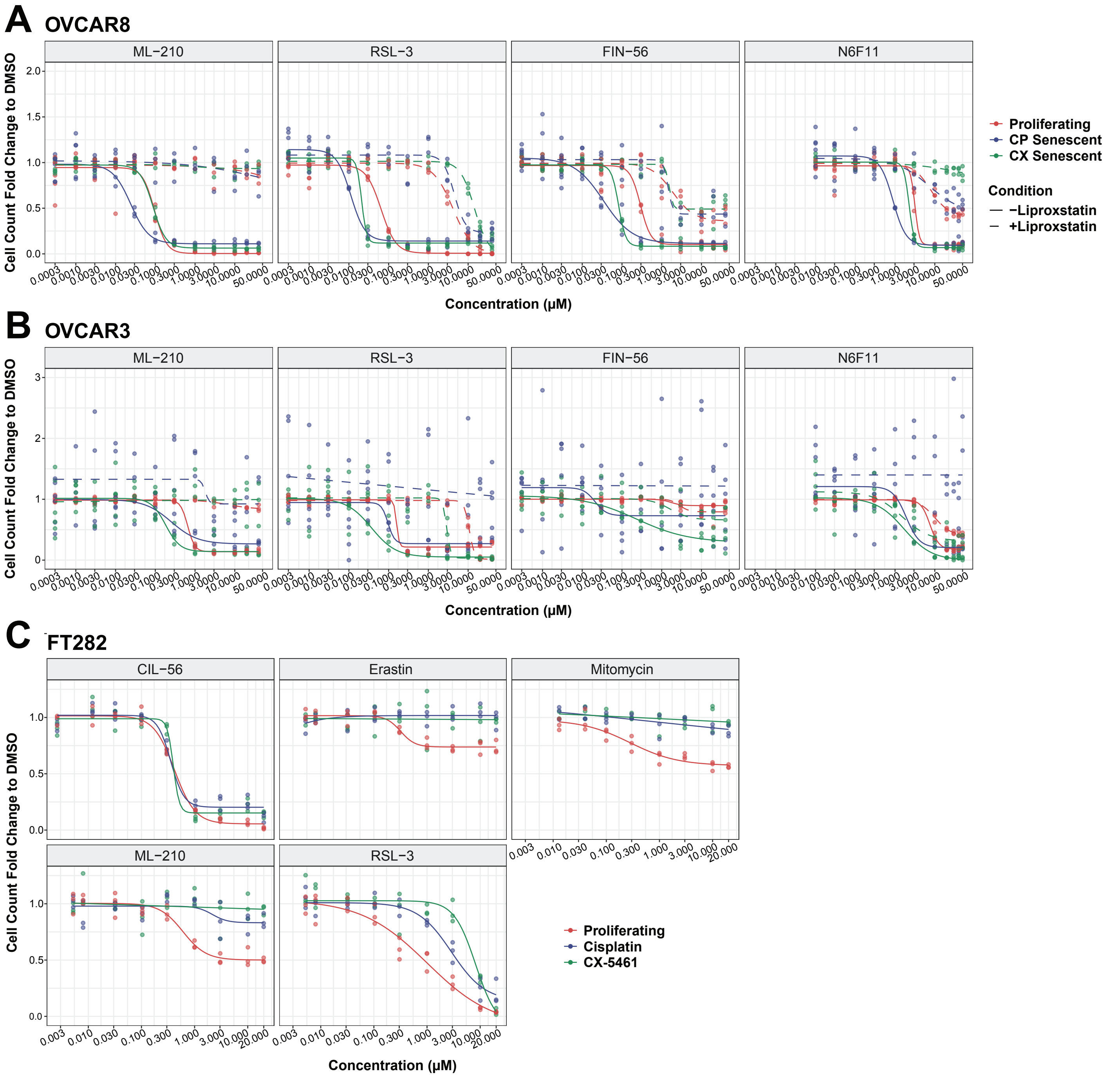

Figure S5. Ferroptosis-inducing drugs show preferential killing of therapy-induced senescent HGSOC cells versus proliferating cells. (A and B) Dose response curves of proliferating or senescent **(A)** OVCAR8 or **(B)** OVCAR3 cells exposed to increasing concentrations of the indicated ferroptosis-inducing drugs. Dashed lines indicate dose response curves with the inclusion of 1 uM liproxstatin. **(C)** Dose response curves of proliferating or senescent FT282 cells exposed to increasing concentrations of ferroptosis-inducing drugs (CIL-56, Erastin, ML-210, RSL3) or mitomycin.

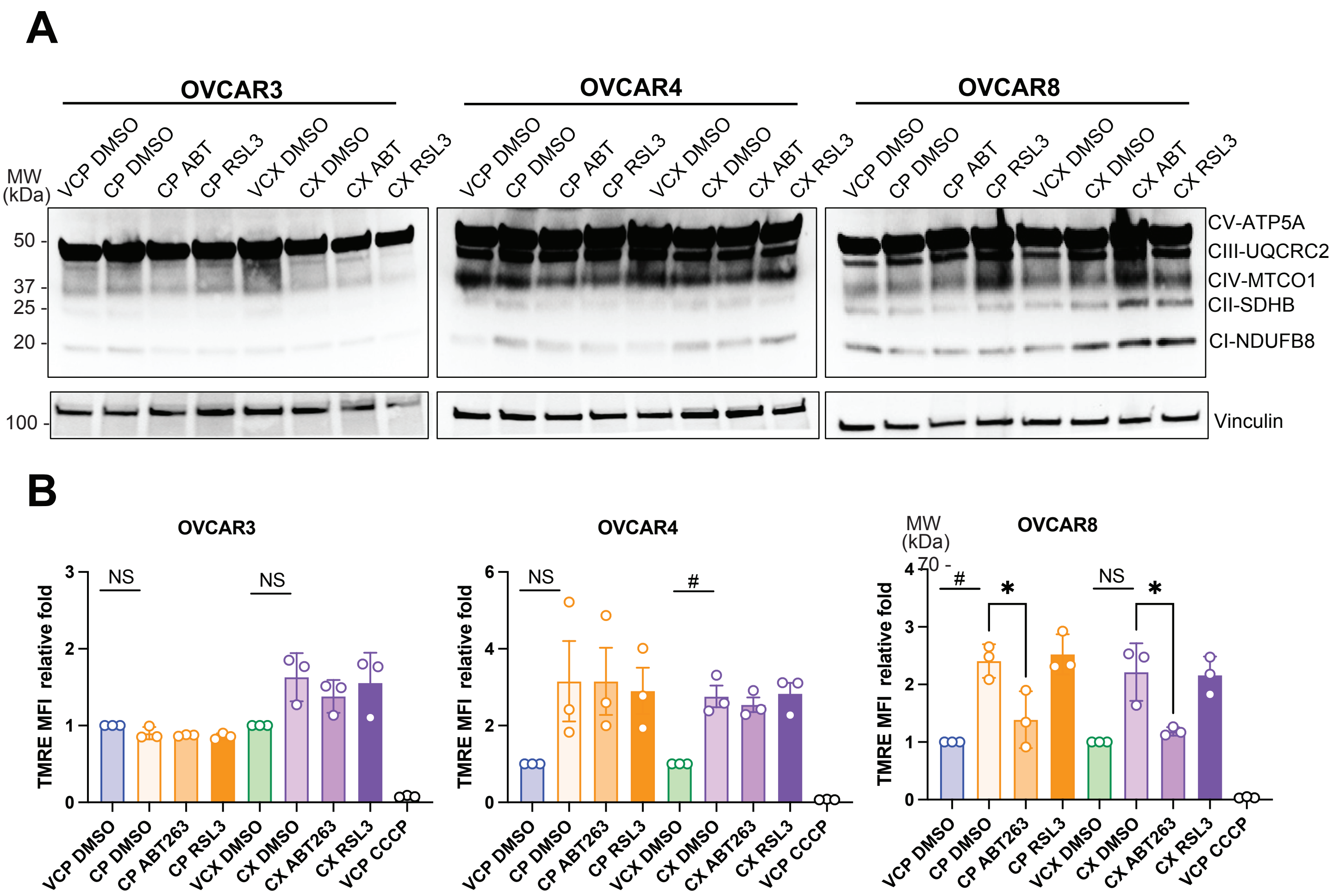

Figure S6. Therapy-induced senescent HGSOC cells show ferroptosis-associated mitochondrial changes. **(A)** Representative Western blots of mitochondrial oxidative phosphorylation complex proteins in OVCAR3, OVCAR4 and OVCAR8 cells exposed to cisplatin (CP) and CX-5461 (CX) and the vehicle controls (VCP and VCX) upon treatment with ABT263 10 μM or RSL3 1 μM for 6 hours. Vinculin was probed as a loading control. **(B)** Relative mitochondrial membrane potential as measured by TMRE median fluorescence intensity (MFI) of drug-treated senescent cells compared to vehicle proliferating controls (VCP DMSO or VCX DMSO). Data are presented as mean ± SEM from n=3 independent experiment. Statistical analysis was performed using one-way ANOVA with a Šídák's multiple comparisons test,*, $p < 0.05$; and one-sample t test compared to the respective vehicle proliferating controls, #, $p < 0.05$; NS = not significant.

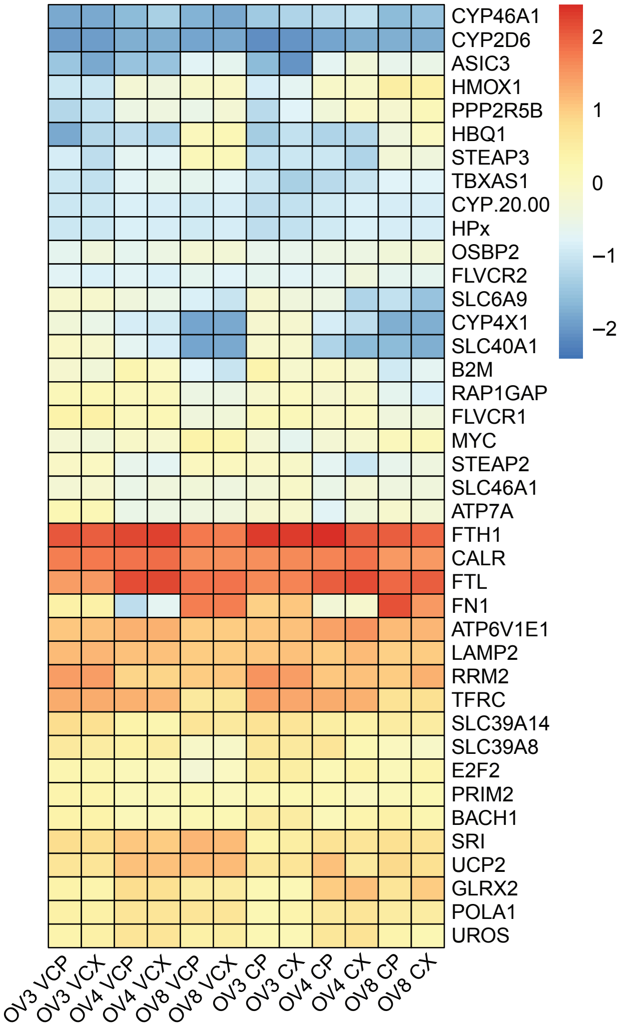

Figure S7. Therapy-induced senescent HGSOC cells do not show marked alterations in expression of iron metabolism genes. Heatmap of the expression of 40 iron metabolism genes from RNAseq data of OVCAR3, OVCAR4 and OVCAR8 cells exposed to cisplatin or CX-5461 or their vehicle controls.

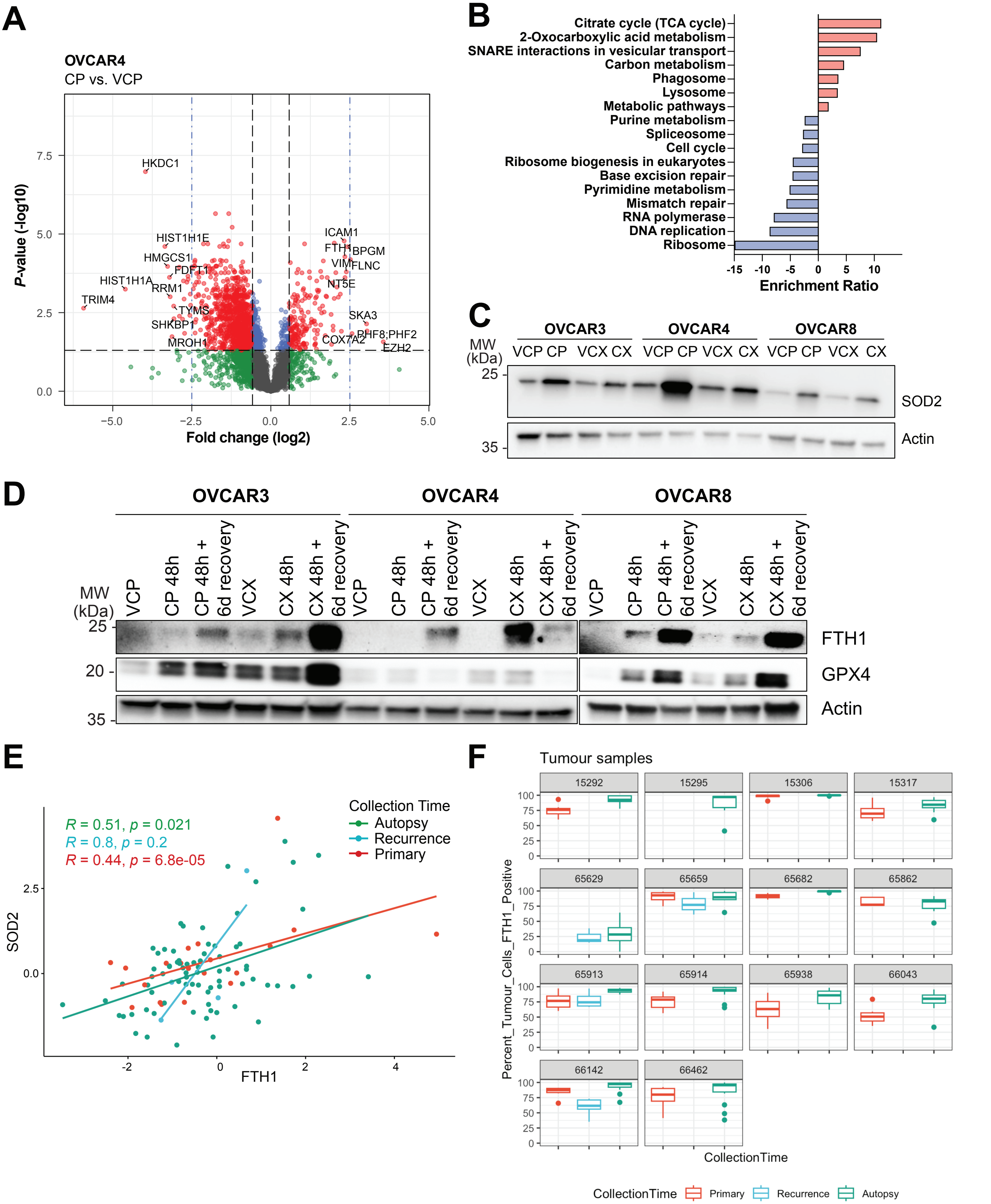

Figure S8. Therapy-induced senescent HGSOC cells show dramatic changes in their proteome. (A) Representative volcano plot showing differentially expressed proteins for OVCAR4 cells exposed to cisplatin or vehicle control. **(B)** Pathway enrichment analysis showing significant biological processes associated with differentially regulated proteins. **(C)** Western blot of SOD2 expression in OVCAR3, OVCAR4 and OVCAR8 cells exposed to cisplatin or CX-5461 or their vehicle controls. Actin was probed as a loading control. **(D)** Western blots of FTH1 or GPX4 expression in OVCAR3, OVCAR4 and OVCAR8 cells exposed to cisplatin or vehicle controls as indicated. **(E)** Correlation plot of FTH1 and SOD2 protein expression in AOCS patient samples collected at the indicated times. **(F)** Box-and-whisker plots showing quantification of FTH1 IHC staining in WT1-positive tumour cells in AOCS patient samples collected at the indicated times.

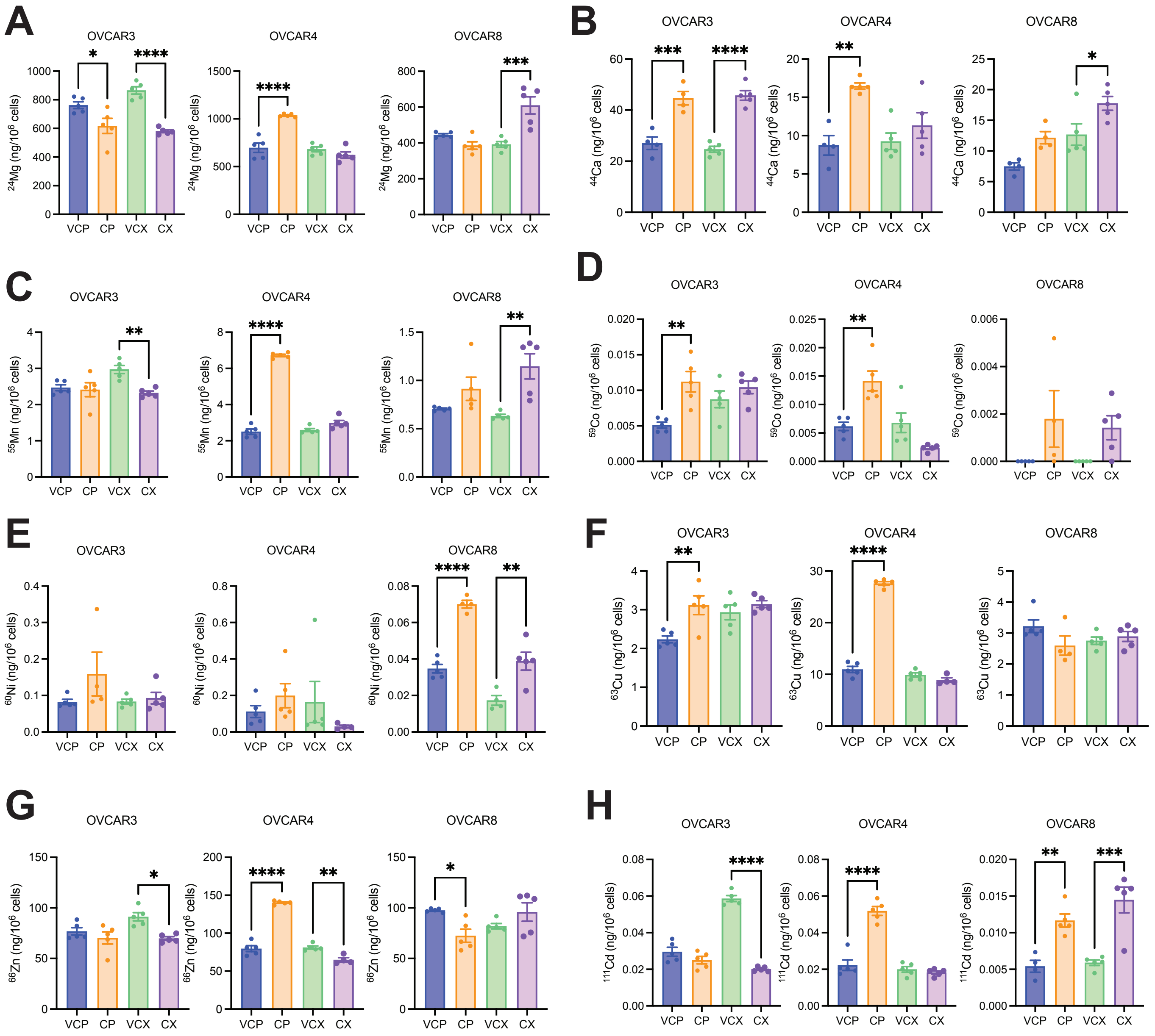

Figure S9. Therapy-induced senescent HGSOC cells display variable changes in intracellular metal ion content. Quantification of metal ion content from ICP-MS analysis of OVCAR3, OVCAR4 and OVCAR8 cells exposed to cisplatin or CX-5461 and their respective vehicle controls, including **(A)** magnesium (^{24}Mg), **(B)** calcium (^{44}Ca), **(C)** manganese (^{55}Mn), **(D)** cobalt (^{59}Co), **(E)** nickel (^{60}Ni), **(F)** copper (^{63}Cu), **(G)** zinc (^{66}Zn) and **(H)** cadmium (^{111}Cd). The data is presented as mean ± SEM from n=4-5 replicates. Statistical analysis was performed using one-way ANOVA with a Šídák's multiple comparisons test. *, $p < 0.05$; **, $p < 0.01$; ***, $p < 0.001$; ****, $p < 0.0001$.

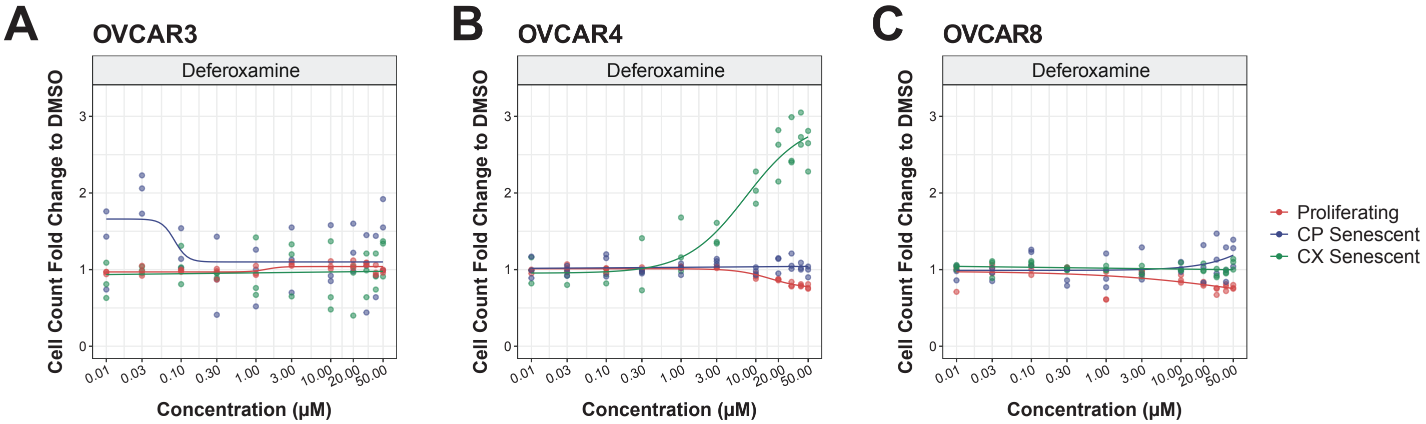

D

OV3 Pro	OV3 CP	OV3 CX	OV4 Pro	OV4 CP	OV4 CX	OV8 Pro	OV8 CP	OV8 CX
1.005	1.236	0.952	0.978	1.03	1.379	0.912	0.98	1.025

Figure S10. Iron chelation promotes survival of therapy-induced senescent HGSOC cells. (A-C) Dose response curves of proliferating or therapy-induced senescent **(A)** OVCAR3, **(B)** OVCAR4 or **(C)** OVCAR8 cells exposed to increasing concentrations of deferoxamine for 24h. **(D)** Quantification of AUC for (A-C).
