# Supplementary material for: Exploiting dysregulated iron homeostasis to eradicate persistent high-grade serous ovarian cancer
Source: Cell Death Discov. 2025 Sep 25;11:423. doi: 10.1038/s41420-025-02716-1 (PMC12462457; doi:10.1038/s41420-025-02716-1)

Figure 2D

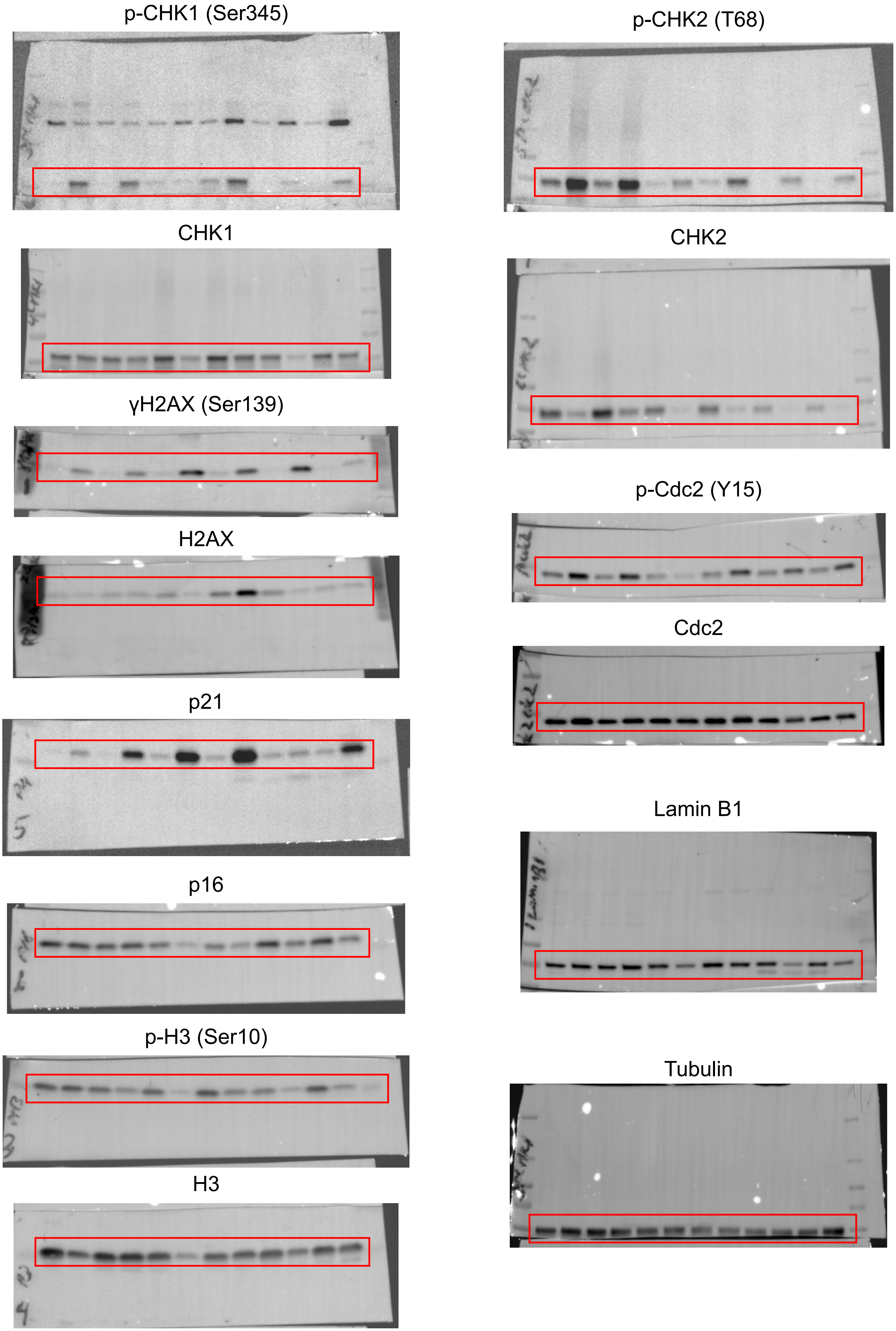
p-CHK1 (Ser345)
p-CHK2 (T68)
CHK1
CHK2
γH2AX (Ser139)
H2AX
p-Cdc2 (Y15)
Cdc2
p21
Lamin B1
p16
p-H3 (Ser10)
Tubulin
H3

Figure 5C

FTH1

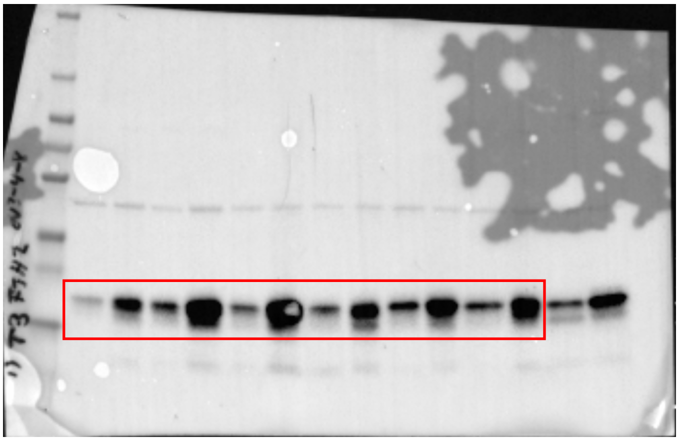

Actin + FTH1

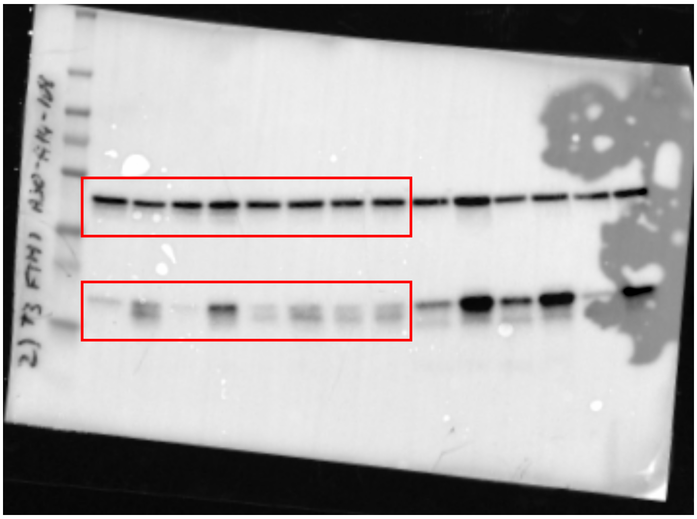

Actin

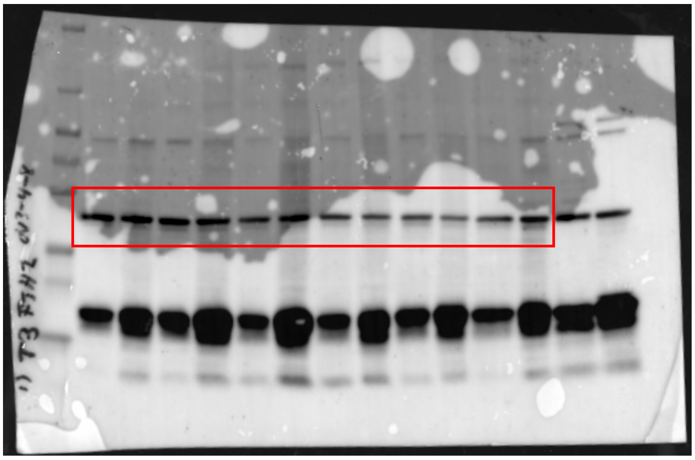

Figure 5F

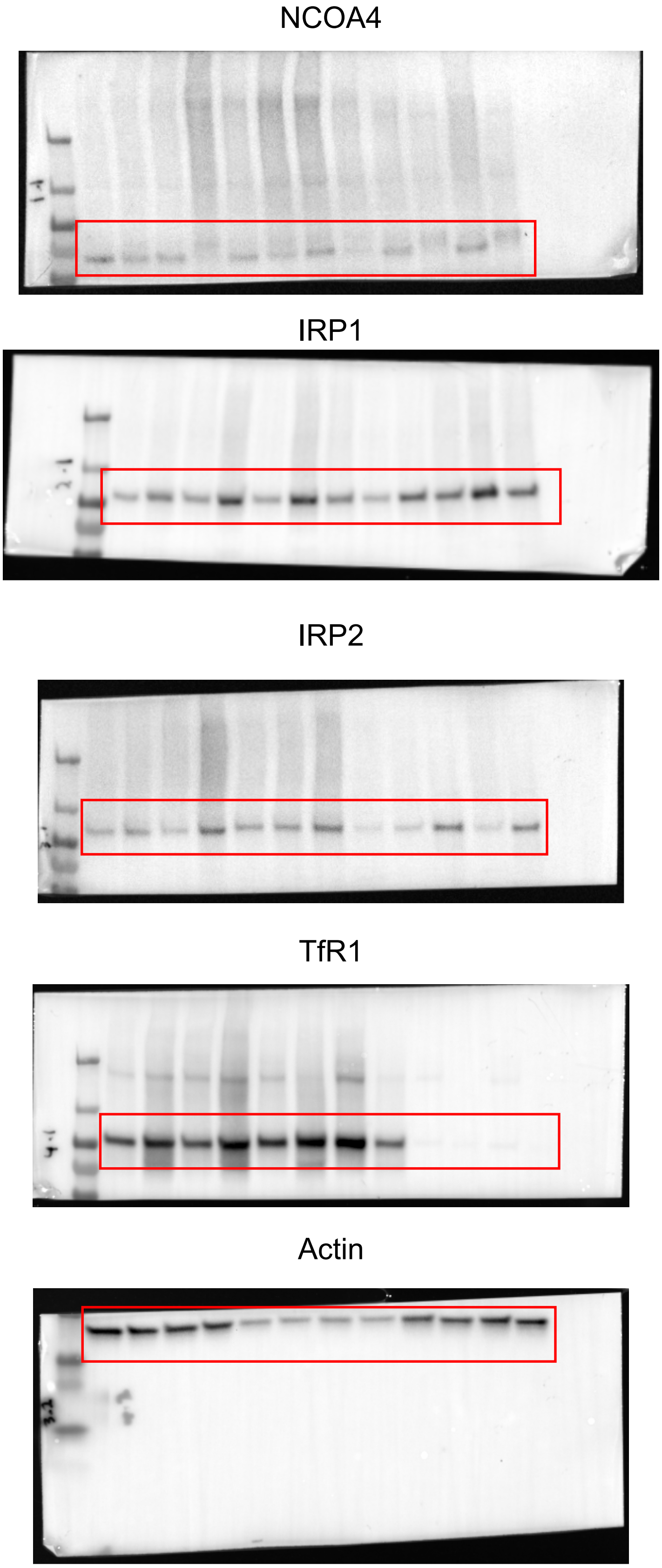

Figure S1C

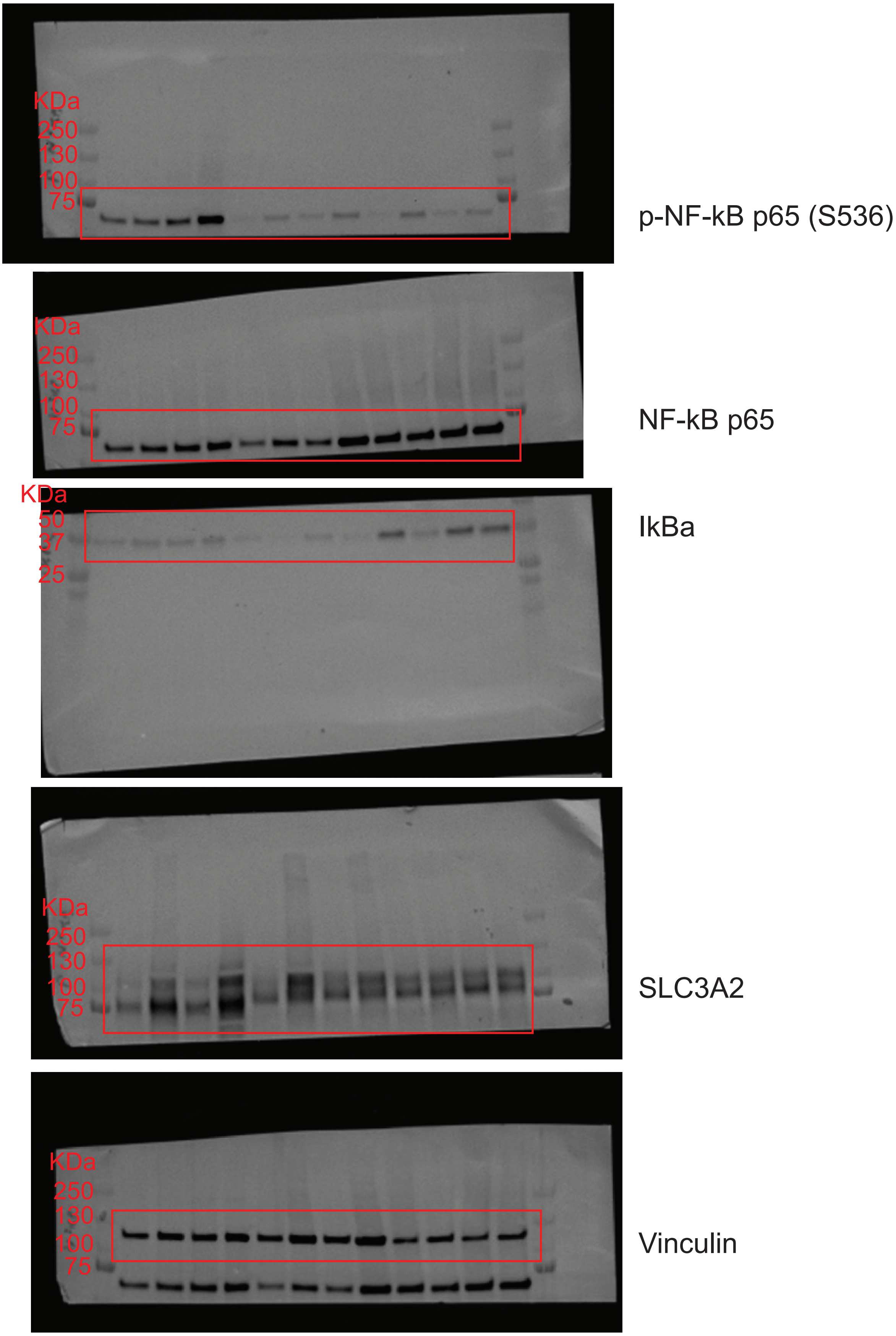
KDa
250
130
100
75
p-NF-kB p65 (S536)
KDa
250
130
100
75
NF-kB p65
KDa
50
37
25
IkBa
KDa
250
130
100
75
SLC3A2
KDa
250
130
100
75
Vinculin

Figure S3A

BCL-XL

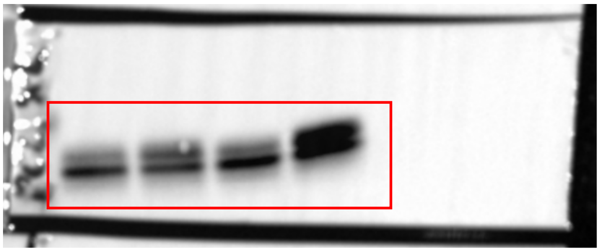

Actin

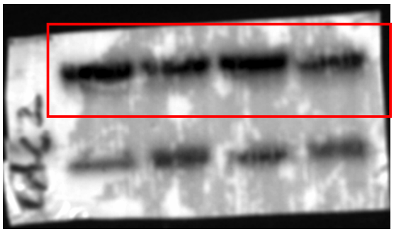

Figure S6A

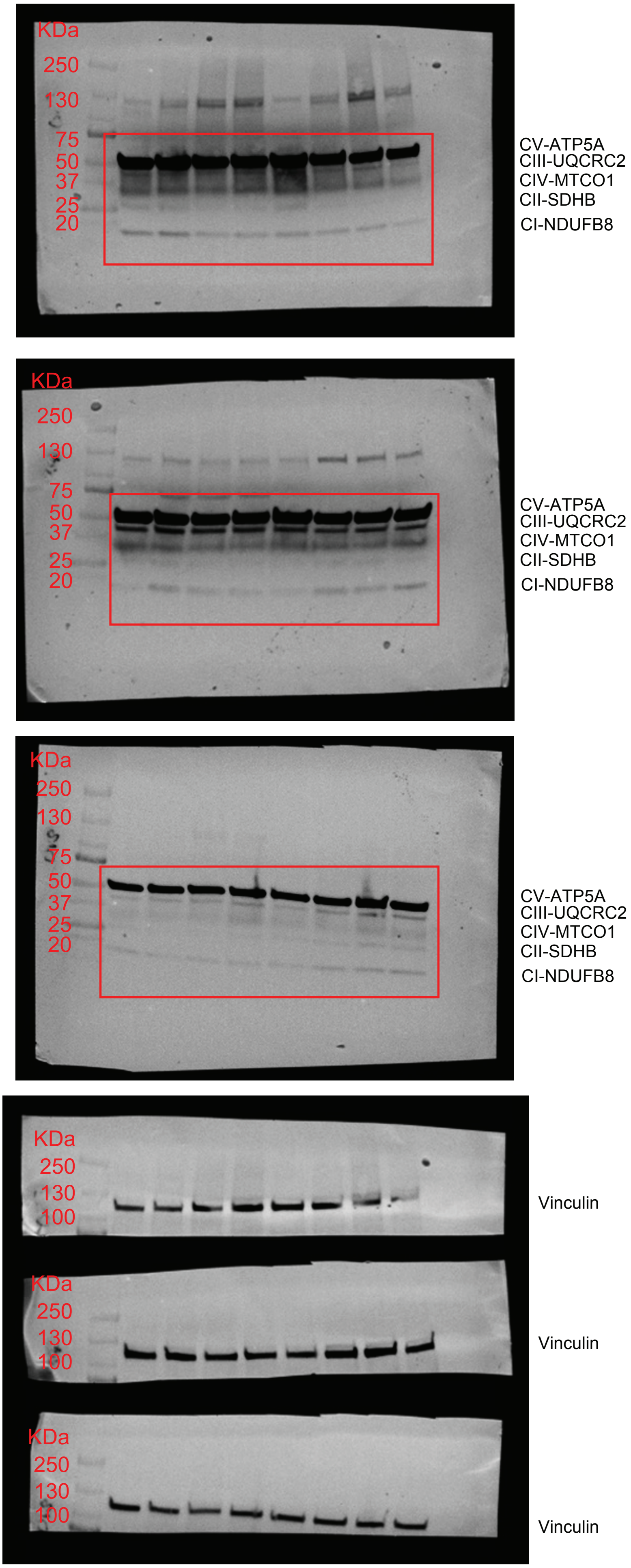
KDa
250
130
75
50
37
25
20
CV-ATP5A
CIII-UQCRC2
CIV-MTCO1
CII-SDHB
CI-NDUFB8
KDa
250
130
75
50
37
25
20
CV-ATP5A
CIII-UQCRC2
CIV-MTCO1
CII-SDHB
CI-NDUFB8
KDa
250
130
75
50
37
25
20
CV-ATP5A
CIII-UQCRC2
CIV-MTCO1
CII-SDHB
CI-NDUFB8
KDa
250
130
100
Vinculin
KDa
250
130
100
Vinculin
KDa
250
130
100
Vinculin

Figure S8C

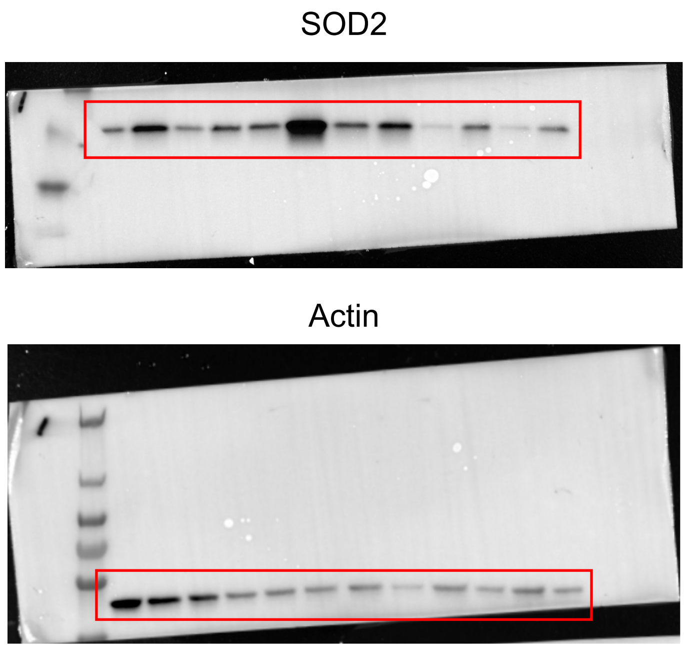

Figure S8D

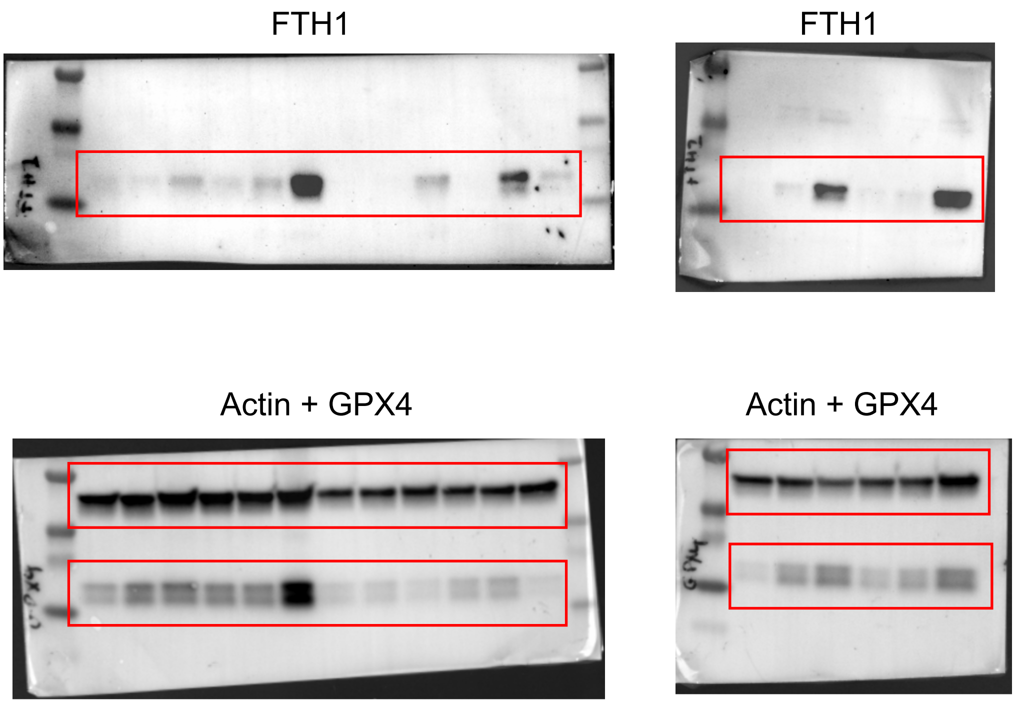

Supplement: Supplementary file 3 — Original Western Blots [file 41420_2025_2716_MOESM3_ESM.pdf]
